# Gd-nanoparticles functionalization with specific peptides for ß-amyloid plaques targeting

**DOI:** 10.1186/s12951-016-0212-y

**Published:** 2016-07-25

**Authors:** Marie Plissonneau, Jonathan Pansieri, Laurence Heinrich-Balard, Jean-François Morfin, Nathalie Stransky-Heilkron, Pascaline Rivory, Pierre Mowat, Mireille Dumoulin, Richard Cohen, Éric Allémann, Éva Tόth, Maria Joao Saraiva, Cédric Louis, Olivier Tillement, Vincent Forge, François Lux, Christel Marquette

**Affiliations:** 1 Laboratoire de Chimie et Biologie des Métaux, Université Grenoble Alpes, CEA Life Science Division, CNRS, 17 rue des Martyrs, 38054 Grenoble Cedex 9, France; 2 Nano-H S.A.S, 38070 Saint Quentin Fallavier, France; 3 Institut Lumière Matière, UMR5306 CNRS Université Claude Bernard Lyon 1, Domaine Scientifique de La Doua, 2 rue Victor Grignard, 69622 Villeurbanne Cedex, France; 4 Université de Lyon, Lyon, France; 5 ISPB Faculté de Pharmacie, 4bis-MATEIS UMR CNRS 5510, 69373 Lyon, France; 6 Centre de biophysique moléculaire, UPR 4301, Rue Charles Sadron CS 80054, 45071 Orléans Cedex 2, France; 7 School of pharmaceutical Sciences, University of Geneva, University of Lausanne Pharmaceutical technology, Quai Ernest-Ansermet 30, 1211 Geneva, Switzerland; 8 Centre for Protein Engineering, Laboratory of Enzymology and Protein Folding Institut of Chemistry, B6 University of Liege Sart Tilman, 4000 Liege, Belgium; 9 Laboratoire de Biochimie et de Biologie Moléculaire, Hospices Civils de Lyon, Lyon, France; 10 Molecular Neurobiology Group, Institute of Biology, Molecular and Cellular, R. Campo Alegre 823, 4150 Porto, Portugal

**Keywords:** Beta-amyloid fibrils, MRI contrast agent, Gadolinium based nanoparticles, Amyloid imaging, Peptide-targeting, Alzheimer’s disease

## Abstract

**Background:**

Amyloidoses are characterized by the extracellular deposition of insoluble fibrillar proteinaceous aggregates highly organized into cross-β structure and referred to as amyloid fibrils. Nowadays, the diagnosis of these diseases remains tedious and involves multiple examinations while an early and accurate protein typing is crucial for the patients’ treatment. Routinely used neuroimaging techniques such as magnetic resonance imaging (MRI) and positron emission tomography (PET) using Pittsburgh compound B, [^11^C]PIB, provide structural information and allow to assess the amyloid burden, respectively, but cannot discriminate between different amyloid deposits. Therefore, the availability of efficient multimodal imaging nanoparticles targeting specific amyloid fibrils would provide a minimally-invasive imaging tool useful for amyloidoses typing and early diagnosis. In the present study, we have functionalized gadolinium-based MRI nanoparticles (AGuIX) with peptides highly specific for Aβ amyloid fibrils, LPFFD and KLVFF. The capacity of such nanoparticles grafted with peptide to discriminate among different amyloid proteins, was tested with Aβ(1–42) fibrils and with mutated-(V30M) transthyretin (TTR) fibrils.

**Results:**

The results of surface plasmon resonance studies showed that both functionalized nanoparticles interact with Aβ(1–42) fibrils with equilibrium dissociation constant (K_d_) values of 403 and 350 µM respectively, whilst they did not interact with V30M-TTR fibrils. Similar experiments, performed with PIB, displayed an interaction both with Aβ(1–42) fibrils and V30M-TTR fibrils, with K_d_ values of 6 and 10 µM respectively, confirming this agent as a general amyloid fibril marker. Thereafter, the ability of functionalized nanoparticle to target and bind selectively Aβ aggregates was further investigated by immunohistochemistry on AD like-neuropathology brain tissue. Pictures clearly indicated that KLVFF-grafted or LPFFD-grafted to AGuIX nanoparticle recognized and bound the Aβ amyloid plaque localized in the mouse hippocampus.

**Conclusion:**

These results constitute a first step for considering these functionalized nanoparticles as a valuable multimodal imaging tool to selectively discriminate and diagnose amyloidoses.

**Electronic supplementary material:**

The online version of this article (doi:10.1186/s12951-016-0212-y) contains supplementary material, which is available to authorized users.

## Background

**Fig. 1:**
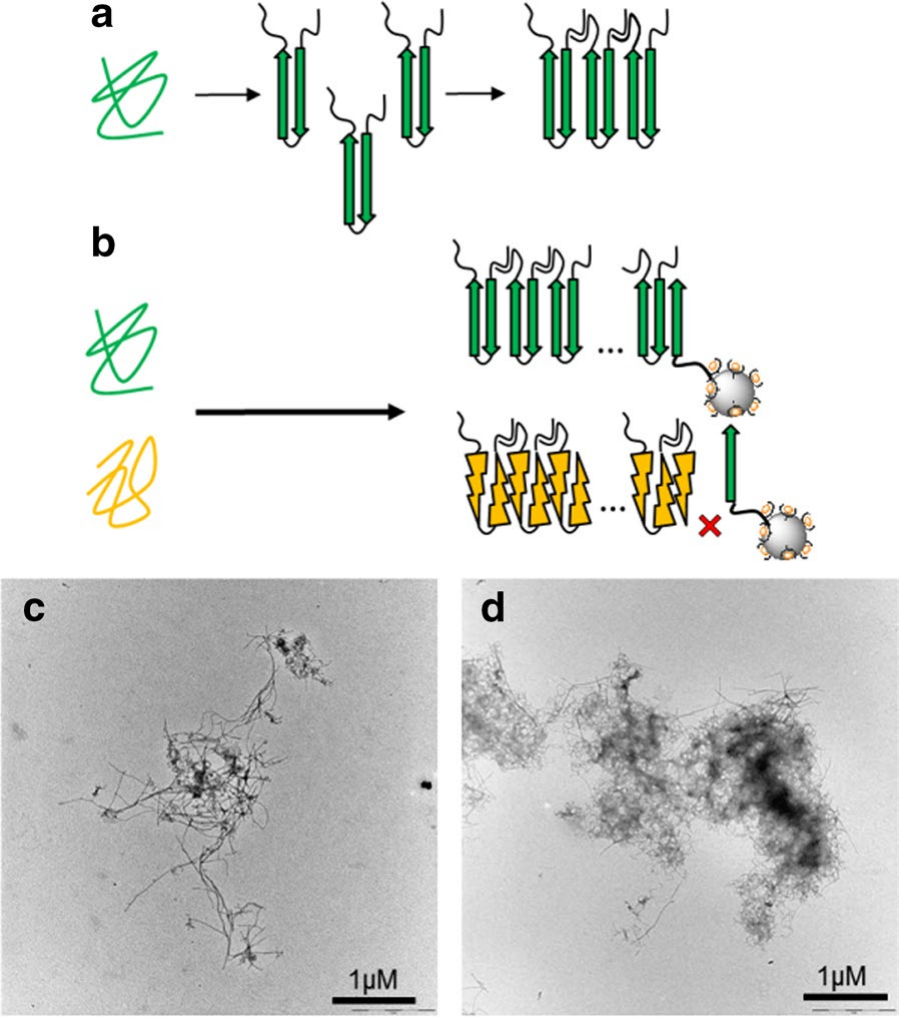
**a** Schematic representation of self-auto-aggregation process leading to insoluble amyloid fibrils formation. **b** Strategy for specific amyloid protein targeting using AGuIX grafted with a selected peptide of Aβ. The nanoparticles are supposed to target the end of Aβ fibrils (represented in *green*) by the selected and grafted peptide, but not to amyloid fibrils of transthyretin (represented in *yellow*). **c** Transmission electronic microscopy pictures of Aβ amyloid fibrils after 7 days of incubation and (**d**) of transthyretin amyloid fibrils formed with V30M mutated protein, at 20 days of incubation

Amyloidoses constitute a wide range of human diseases which are related to the conversion of soluble proteins into well-organized and insoluble fibrillar deposits, due to protein misfolding and self-association into amyloid aggregates enriched with cross-β sheet structures [1] (Fig. 1a). There are approximately 40 disorders associated with amyloid fibril formation and deposition in the extracellular space of various organs and tissues. They include neurodegenerative diseases (e.g. Alzheimer’s disease, AD), systemic amyloidoses (e.g. familial amyloid polyneuropathy, FAP) and localized amyloidoses (e.g. type II diabetes). Each disorder has a distinct clinical profile and is associated with the aggregation of a predominant peptide or protein [2], i.e. amyloid-β peptide (Aβ) in AD, transthyretin (TTR) in FAP, or amylin in type II diabetes. Currently, the diagnosis of amyloidoses is established from the results of multiple invasive examinations [3, 4], and is complicated by the involvement of several organs, and by the fact that symptoms are unspecific. In recent years, amyloidoses attracted considerable interest from scientists in different disciplines, resulting in a better understanding of their pathogenesis and of the molecular mechanisms of amyloid formation. This knowledge has led to important advances useful for designing new tools to achieve early and reliable diagnosis of these pathologies.

Among amyloidosis, the major cause of dementia in the elderly population, Alzheimer’s disease is the most common age-related neurodegenerative disease. The number of people living with dementia worldwide is estimated at 44 million, and is set to almost double by 2030 and more than triple by 2050 [5]. Establish AD diagnosis needs long and exhaustive cognitive test, completed by neuro-imaging examination in order to be differentiated from other causes of dementia: vascular dementia, dementia with Lewis bodies, Parkinson’s disease with dementia, frontotemporal dementia and reversible dementias. The neuropathological hallmarks of AD include extracellular plaques of Aβ peptide, intracellular neurofibrillary tangles and dystrophic neurites, which constitute the final markers attesting definitively of the disease. These histopathologic lesions are restricted to selective brain regions involved in memory and language, i.e. the hippocampus and the cortex [6]. The progressive aggregation of Aβ peptides into plaque structures is one of the critical events leading to the progressive dismantling of synapses, neuronal circuits and networks [7–9], and to neurodegeneration [10, 11]. Interestingly, the appearance of amyloid plaques occurs years before the appearance of cognitive symptoms [12, 13] and is considered to be the distinct hallmark of early onset of the disease activating the sequential lesion events. Therefore, the in vivo detection and quantification of amyloid species within the brain of patients at risk constitutes a promising strategy for the early diagnosis and treatment of Alzheimer’s disease.

Currently, magnetic resonance imaging (MRI) is the preferred neuroimaging examination for AD as it allows an accurate measurement of brain structures’ volume, although a decrease of this latter appears to be a nonspecific and late feature of cerebral vascular diseases’ progression [14]. Thus, studies are under way to develop specific imaging markers for different types of dementia, including markers of senile plaques for very-high-field MRI and positron emission tomography (PET) [15]. A better characterization of the amyloid plaque presence and load in the brain can be also expected from imaging approaches using amyloid ligands as imaging agents [16]. One of the most extensively used is the Pittsburgh compound B (PIB, [^11^C]2-(4′-methylaminophenyl)-6-hydroxybenzothiazole), which binds with high affinity to any amyloid fibrils [17–21] and displays a PET signal in vivo, and correlates strongly with the amyloid burden [20]. Although several PET probes are currently under clinical investigation, and preliminary results indicate that these tracers cannot differentiate between amyloid plaques at various stages and of different nature [17, 19–23]. Indeed, irrespective of the neurodegenerative disease investigated, none of these studies have mapped Aβ pathologies in sufficient details to allow a quantitative correlation between the PET signal and the amyloid burden in different regions of the brain. Thus, novel non-invasive Aβ binding substances suitable for in vivo imaging, with high protein specificity and detection sensitivity, need to be elaborated.

The development of multifunctional nanoparticles represents a breakthrough in medical imaging since these nanoobjects can meet the requirements of several imaging techniques such as MRI, optical imaging or single photon emission CT (SPECT)/PET scintigraphy while offering excellent resolution and sensitivity. For instance, functionalization of such nanoparticles with specific biovectors allows their active targeting to tumors leading to a more accurate diagnosis [24]. Likewise, small Aβ peptides were coupled to gold nanoparticles in order to inhibit Aβ fibrillogenesis [25] or to ultrasmall superparamagnetic iron oxide nanoparticles in order to design contrast agents for Aβ plaque MRI imaging [26].

Recently, sub-5 nm gadolinium-based nanoparticles [27, 28] (AGuIX) have been developed that are suitable for multimodal detection: (1) in MRI, DOTAGa(Gd^3+^)-grafted AGuIX nanoparticles led to significantly higher positive contrast when compared to classical molecular agents such as DOTAREM^®^ [27, 29, 30]; (2) SPECT/PET can be carried out following radiolabeling; and (3) in vivo near infrared fluorescence imaging can be performed following the grafting of an appropriate fluorophore. Such multimodal imaging platform, gathering multi-detection possibilities, allows achieving, in vivo, a sensitive and reliable imaging. Furthermore, their functionalization with RGD peptides was realized in order to target and detect tumors [30, 31]. Due to their small size and biodegradability, AGuIX are efficiently eliminated from the body through renal clearance with no evidence of toxicity [32].

With the aim to develop a sensitive imaging tool which target amyloid fibrils and able to discriminate between different protein amyloid aggregates, we have focused on Aβ(1–42) fibrils as a proof of concept for amyloidosis. To this end, AGuIX nanoparticles were grafted with two small peptides derived from the sequence of Aβ(1–42). The peptide KLVFF (corresponding to the hydrophobic core short Aβ(16–20) fragment) which is crucial for the formation of the β-sheet structures [33, 34] and binds to the full-length Aβ peptide via atypical antiparallel β-sheet motif [35, 36], and the peptide LPFFD which binds to the central hydrophobic region of Aβ [37]. The rationale behind our investigation is that thanks to their capacity to co-aggregate with Aβ(1–42), KLVFF and LPFFD grafted on AGuIX are expected to target specifically the fibril ends of Aβ(1–42) deposits (Fig. 1b), their interaction is reported by fluorescence detection of a near infrared fluorophore (cyanine 5.5) also chemically grafted on AGuIX.

## Results and discussion

In this work, we have optimized the functionalization of AGuIX nanoparticles with peptides in order to selectively target Aβ(1–42) fibrils and detect amyloid plaque in AD animal tissue. AGuIX synthesis were firstly modified and optimized by addition of a PEG chain for LPFFD and KLVFF grafting optimization, the two selected Aβ(1–42) pentapeptides. Then, AGuIX@PEG@LPFFD and AGuIX@PEG@KLVFF were labeled with the near infrared fluorescent compound cyanine 5.5, allowing in vitro and ex vivo experiments. The binding specificity was tested using amyloid fibril: (1) Aβ amyloid (1–42) fibrils, implicated into plaque amyloid burden in AD, and (2) Val->30->Met mutated transthyretin (V30M-TTR) fibrils [38, 39], one of the more than 80 identified mutations on TTR as causative gene abnormality in FAP [40]. The efficiency of the selective interaction was also compared with the PIB-binding to each amyloid fibril. Finally, the specificity of recognition of Aβ-grafted nanoparticles was achieved by immunohistochemistry experiments on brain sections of Alzheimer’s disease mouse model.

### Nanoparticles functionalization with targeting peptides

AGuIX nanoparticles (Nps) were synthetized by an original top–down process as previously described [29]. This synthesis led to the formation of Nps displaying a hydrodynamic diameter of 2.7 ± 0.7 nm and an average molecular weight of 8.5 ± 1.0 kDa as judged by DLS and HPLC respectively (Additional file 1). The skeleton of the nanoparticles is composed of a polysiloxane network containing free amino functions obtained by the use of APTES ((3-Amino)propyltriethoxy silane) precursor during sol/gel process. The primary amine of APTES allows covalent grafting of about ten DOTAGa (Gd^3+^) chelates per particle through amide bond formation resulting from the reaction of the amines with the anhydride function. Small chains of polyethylene glycol diacid (PEG) were added to nanoparticles with one end to be grafted covalently to the nanoparticle, the other remaining available for peptide coupling (Additional file 1). The nanoparticles surface modification was monitored and characterized by complementary techniques, including Fourier Transform Infrared spectroscopy (FTIR) and measurement of the ζ-potential (Additional file 1). Altogether, the results clearly indicated that PEG chains have been grafted to the nanoparticles, leading to about 5 carboxylic acid functions per Np. Therefore, these AGuIX@PEG are suitable for peptides coupling (Additional file 1).

**Fig. 2:**
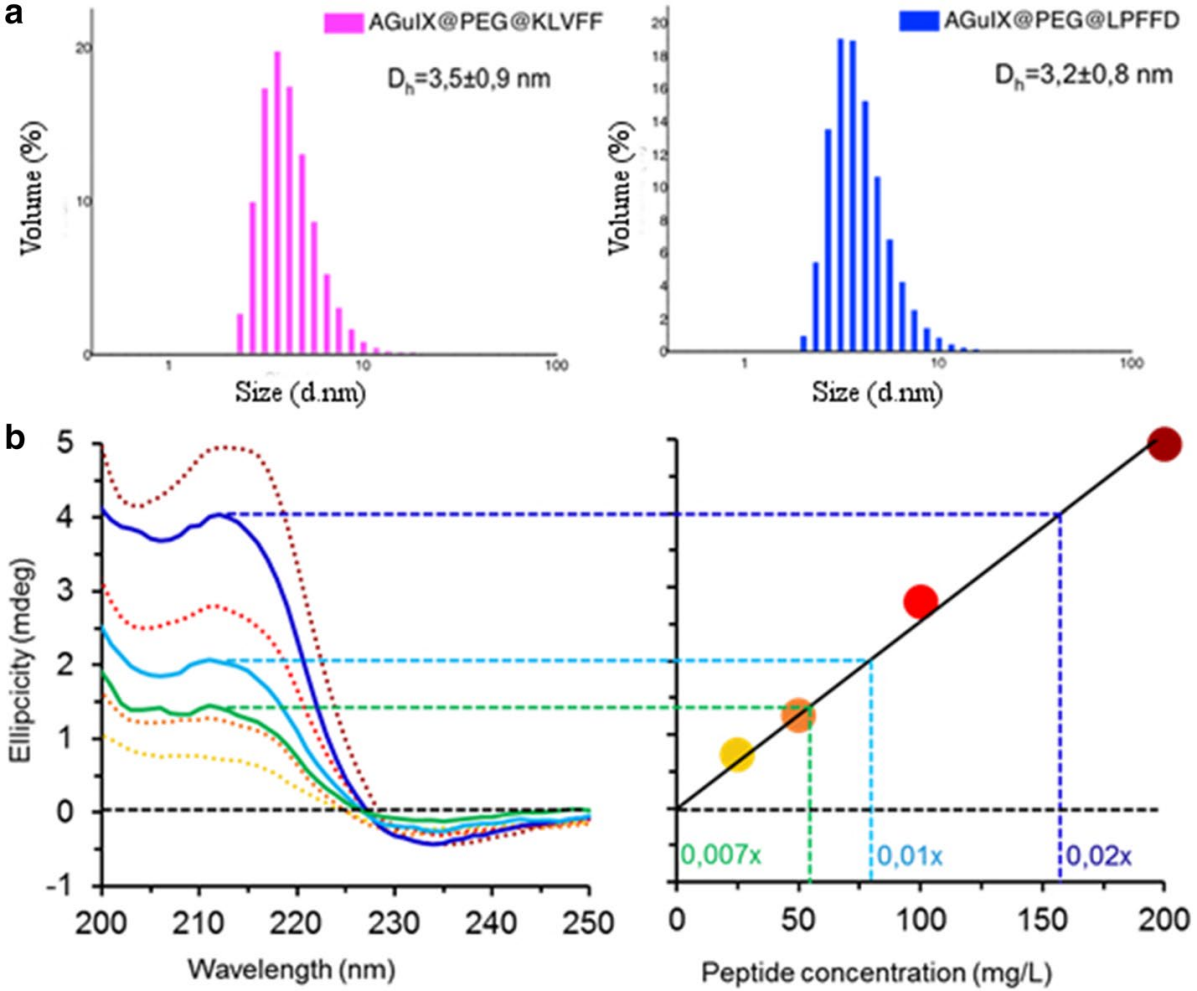
Amyloid peptide-targeted AGuIX characterization: **a** hydrodynamic diameter measurements of AGuIX@PEG@KLVFF (*pink*) and AGuIX@PEG@LPFFD (*blue*) by dynamic light scattering measurements (λ = 532 nm). **b** Quantification of the number of peptides grafted on AGuIX^®^ nanoparticles. Circular dichroism signal of free KLVFF at 25, 50, 100, 200 mg L^−1^ at 212 nm (*dotted lines*) were reported on standard curve with reporting ellipcicity of peptide on nanoparticles in concentration function. To quantify the number of KLVFF grafted per nanoparticle, various dilution (from 50 to 150 times) (*continuous lines*) were measure and reported on the graph with a number of peptide correspondence

KLVFF and LPFFD were grafted to nanoparticles by amide formation thanks to classical carbodiimide chemistry between their N-terminal and the carboxylic acid function of the AGuIX@PEG (Additional file 1). Dynamic light scattering measurement (Fig. 2a) showed a slight increase of the Nps hydrodynamic diameter after peptide grafting: 3.2 ± 0.8 nm for the AGuIX@PEG@LPFFD and 3.5 ± 0.9 nm for the AGuIX@PEG@KLVFF compared to 2.9 ± 0.9 nm for the AGuIX@PEG. The shifts observed in relaxometry (Table 1), HPLC (Additional file 1), and DLS (Fig. 2a) are also in accordance with an efficient grafting of the peptides on AGuIX@PEG. The number of Aβ-peptides grafted to the nanoparticles was evaluated by far UV-circular dichroism at 1.6 ± 0.2 LPFFD-peptide per AGuIX@PEG and 2.25 ± 0.10 KLVFF-peptide per AGuIX@PEG (Fig. 2b). In parallel, elementary analyses were performed on the vectorized nanoparticles and confirmed the presence of about two peptides per nanoparticle (Additional file 1). These findings were validated by the quantification of free amine in the nanoparticles using the trinitrobenzene sulfonic acid (TNBS) method [41] (Additional file 1).

**Table 1:** Transversal and longitudinal relaxivities of the nanoparticles and the molecular contrast agent DOTAREM^®^, and the ratio between their transversal and their longitudinal relaxivities at 60 MHz and 37 °C

	AGuIX	AGuIX@PEG	AGuIX@PEG@LPFFD	AGuIX@PEG@KLVFF	DOTAREM^®^
r_1_ (s^−1^ mM^−1^)	10	12	13.2	14.1	3.4
r_2_(s^−1^ mM^−1^)	13.6	18.7	20.1	23.0	4.8
r_2_/r_1_	1.4	1.6	1.5	1.6	1.4

The quality of both AGuIX@PEG@LPFFD and AGuIX@PEG@KLVFF as MRI contrast agent was verified according to their relaxivity values. Both longitudinal (r1) and transversal (r2) relaxivities increased after peptide grafting. These high values are due to the important molecular weight of the Nps and to the rigidity induced by the Nps skeleton, leading to an increase of the rotation correlation time [42, 43]. Thus, the AGuIX@PEG showed a r1 = 12.0 s^−1^ mM^−1^ and r2 = 18.7 s^−1^ mM^−1^, when these values rose for the AGuIX@PEG@LPFFD (r1 = 13.2 s^−1^ mM^−1^ and r2 = 20.1 s^−1^ mM^−1^) and for the AGuIX@PEG@KLVFF (r1 = 14.1 s^−1^.mM^−1^ and r2 = 23.0 s^−1^ mM^−1^). The r2/r1 ratios tend to one (Table 1). These values are significantly higher than those of the molecular contrast agents (DOTAREM^®^: r1 = 3.4 s^−1^ mM^−1^ and r2 = 4.8 s^−1^ mM^−1^ at 60 MHz) confirming the MRI positive contrast imaging feature of AGuIX nanoparticles [44] (Table 1). These measures indicated that the two distinct nanoparticles grafted with specific Aβ peptide have been obtained while maintaining appropriate MRI characteristics.

Cyanine 5.5 was chosen as dye-label for AGuIX@PEG@LPFFD and AGuIX@PEG@KLVFF, to allow biophysics and ex vivo experiments due to absorption and emission wavelengths in the near infrared (λ_abs.max_ = 675 nm and λ_em.max_ = 695 nm). The grafting yield was estimated by comparing the Cy5.5 fluorescence intensity before and after purification and led to an amount of 1 dye for 170 Np and 1 dye for 200 Np for AGuIX@PEG@LPFFD@Cy5.5 and AGuIX@PEG@KLVFF@Cy5.5 respectively (Additional file 1).

In order to envisage these functionalized nanoparticles as a safety contrast agent for a translation to in vivo, especially following the addition of small peptides which could introduce cytotoxicity changes of the nanoparticles [45], innocuousness of functionalized nanoparticles has been assessed against neuronal cells. Cytotoxicity assays were performed using the viability test of MTT (3-(4,5-dimethylthiazol-2-yl)-2,5-diphenyltetrazolium bromide) [46] on SH-SY5Y, and the results showed that AGuIX@PEG@LPFFD@Cy5.5, AGuIX@PEG@KLVFF@Cy5.5 and AGuIX@PEG, do not exhibit any toxic effects on cells (Additional file 1). Indeed, more than 80 % of cells were alive after 1 h of incubation in the presence of nanoparticles corresponding to Gd^3+^ concentrations between 0.5 and 2 mM in Gd^3+^. It is also interesting to note that even at higher concentration (5 mM Gd^3+^) more than 75 % of cells were still alive. Previous biocompatibilities assays showed no adverse effects upon systemic AGuIX administration in healthy rodent [32] and those bearing melanoma, carcinoma [47] or gliosarcoma [48], and biodistribution studies indicated their renal excretion within few hours [32].

### Aβ peptide grafted to AGuIX target specifically Aβ(1–42) fibrils

Formation of amyloid fibrils were obtained by incubation of Aβ(1–42) peptide and V30M-TTR protein under conditions favorable to aggregation in 3 and 10 days respectively. The final state of amyloid-fibril formation is controlled by transmission electron microscopy as illustrated on Fig. 1c and d: Aβ(1–42) fibrils are at least 2 µm long with a wide of 10–15 nm (Fig. 1c), whereas the V30M-TTR amyloid fibrils form bundles of about several µm long and 20 nm wide (Fig. 1d).

**Fig. 3:**
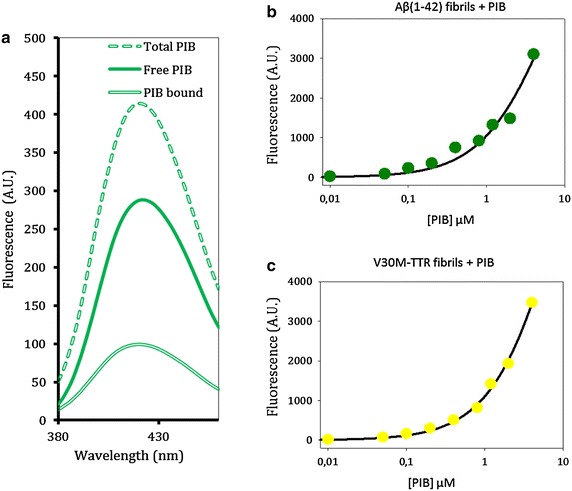
Spectrofluometry measurement of PIB binding to Aβ (1–42) fibrils or V30M-TTR fibrils following partition experiments (λex = 350 nm and λem = 420 nm). **a** 2 µM of PIB was added to 20 µM of amyloid fibrils (*dotted line*) and after 60 min., a pellet with fibrils and PIB bound (*double lines*) was separated from a supernatant with PIB alone (*solid line*). (**b**, **c**) Binding plots by fluorescence response at 420 nm of PIB bound on Aβ(1–42) fibrils (B) or V30M-TTR fibrils (**c**) increasing concentration of PIB, fitted as Michaelis model (1:1)

In order to define an amyloid interaction “gold” standard, K_d_ values were evaluated using the PIB marker with Aβ(1–42) and V30M-TTR fibrils by partition experiments. Following incubation of PIB with Aβ(1–42) or V30M-TTR fibrils, a first measure of the total fluorescence present in the mixture was realized as the total amount of PIB (Fig. 3a, dashed line). Then after centrifugation of the mixture, the PIB fluorescence signal associated to the pellet was measured (Fig. 3a double line), indicating thereof the PIB bound to the fibrils. Similar measure was performed with the supernatant allowing the evaluation of the unbound fluorescence (Fig. 3a, solid line). Experiments were performed with a range of concentrations of PIB (0.01–4 µM) and the results of fluorescent measurements associated with Aβ(1–42) (Fig. 3b) and V30M-TTR (Fig. 3c) fibrils were plotted. The fit of the experimental data with the Michaelis binding model (1:1) displayed K_d_ values equal to 6 µM for Aβ(1–42) fibrils (Fig. 3b) and 10 µM for V30M-TTR fibrils (Fig. 3c). These observations confirm that PIB binds different types of amyloid fibrils making it a general amyloid marker without protein discrimination (Fig. 3b, c). Similar experiments were then performed with AGuIX@PEG@LPFFD@Cy5.5 and AGuIX@PEG@KLVFF@Cy5.5, on Aβ(1–42) fibrils, by measuring the related-Cy5.5 fluorescence. For both nanoparticles, no fluorescence has been detected associated with fibrils (data not shown). Although partition method was adapted for the evaluation of the interaction of PIB marker with fibrils, these latter results indicate that the amyloid fibrils interaction-tests, with both functionalized nanoparticles, displayed certainly a lower detection sensitivity.

Therefore the ability of AGuIX@PEG@LPFFD@Cy5.5 and of AGuIX@PEG@KLVFF@Cy5.5 to target amyloid fibrils was tested using surface plasmon resonance (SPR). This technique allows the observation of interaction in real-time on a sensor chip functionalized with 800 Resonance Unit (R.U.) of V30M-TTR fibrils or 1200 R.U. of Aβ(1–42) fibrils.

**Fig. 4:**
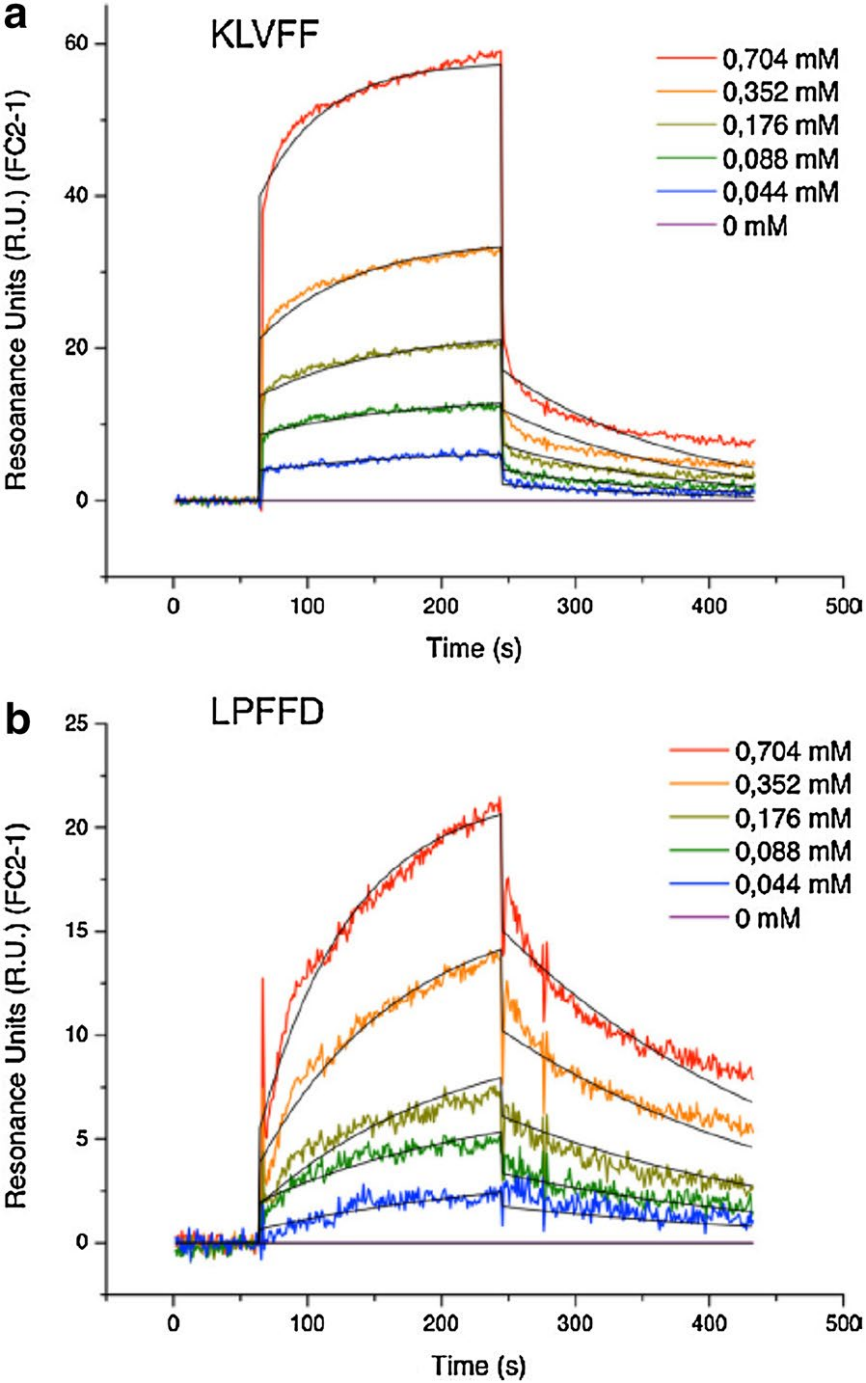
Interaction of KLVFF (**a**) and LPFFD (**b**) peptides at several concentrations with Aβ(1–42) fibrils measured by SPR. The sensorgrams obtained (*colored curves*) were fitted thanks to the Biaevaluation^®^ software with the Langmuir 1:1 model (*black curves*)

As KLVFF and LPFFD were described to interact with monomer forms of Aβ [37, 49], we ensured first that they interact with Aβ(1–42) fibrils. Then SPR experiments were carried out with peptide alone, to determine their affinity onto Aβ(1–42) fibrils (Fig. 4a, b). These results indicate that both selected peptides recognize and interact with Aβ(1–42) fibrils. The K_d_ calculated with the Biaevaluation^®^ software were 393 ± 23 µM for KLVFF and 317 ± 48 µM for LPFFD.

**Fig. 5:**
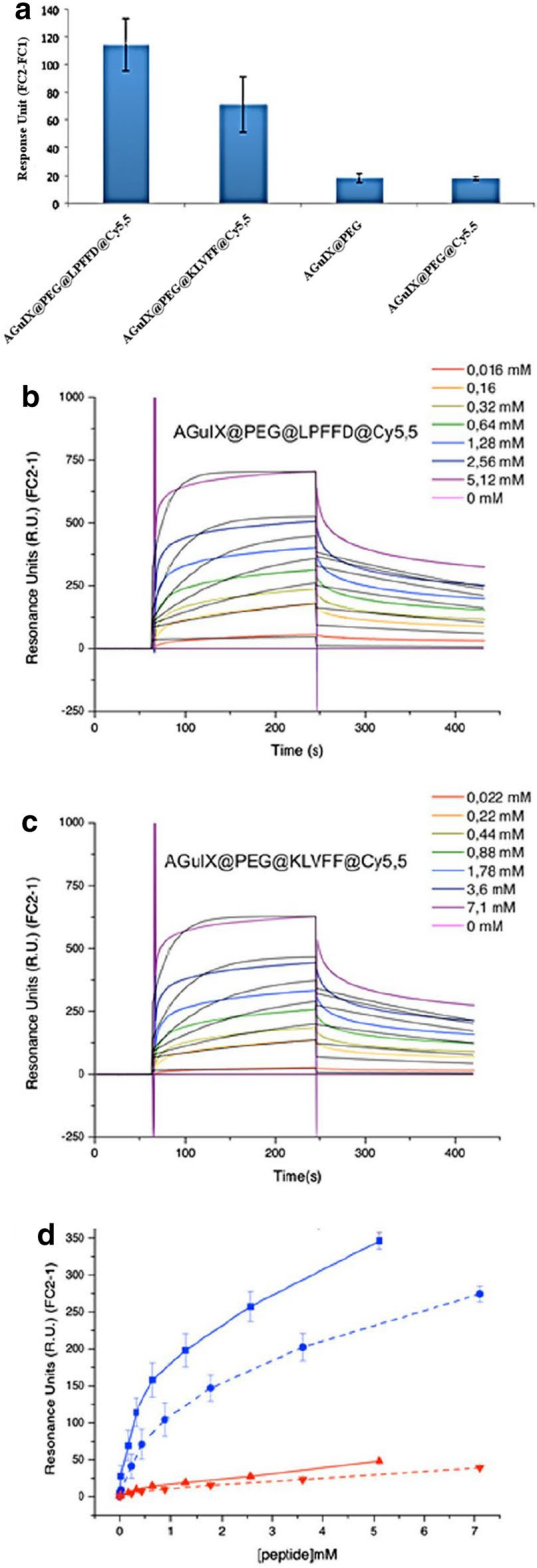
Interaction of functionalized nanoparticles for Aβ(1–42) fibrils or V30M-TTR fibrils assessed by SPR experiments. **a** Remaining signal of AGuIX@PEG, AGuIX@PEG@Cy5.5, AGuIX@PEG@LPFFD@Cy5.5 and AGuIX@PEG@KLVFF@Cy5.5 at 2.5 mM in Gd^3+^ on Aβ(1–42) fibrils. The R.U. were measured 141 s after the end of the analyte injection. **b, c** The sensorgrams (*colored curves*) obtained for AGuIX@PEG@LPFFD@Cy5.5 (**b**) and for AGuIX@PEG@KLVFF@Cy5.5 (**c**) on Aβ(1–42) fibrils were fitted thanks to the Biaevaluation^®^ software with the Langmuir 1:1 model (*black curves*). **c** Remaining signal of AGuIX@PEG@LPFFD@Cy5.5 (*plain curves*) and AGuIX@PEG@KLVFF@Cy5.5 (*dashed curves*) on Aβ(1–42) fibrils (*blue*) and V30M-TTR fibrils (*red*). The R.U. values plotted on the graph correspond to the measures at the time-point 141 s after the end of nanoparticles injection

Thereafter, to insure that the pegylation step of the Nps and the cyanine dye grafting did not induced unexpected binding, the number of R.U. remaining 141 s after the end of the nanoparticles injection was compared to those obtained with AGuIX@PEG, AGuIX@PEG@Cy5.5, AGuIX@PEG@LPFFD@Cy5.5 and AGuIX@PEG@KLVFF@Cy5.5 at 2.5 mM in Gd^3+^ on Aβ(1–42) fibrils. As illustrated by histograms in Fig. 5a, while both AGuIX@PEG@LPFFD@Cy5.5 and AGuIX@PEG@KLVFF@Cy5.5 interact with Aβ(1–42) fibrils, AGuIX@PEG and AGuIX@PEG@Cy5.5 do not interact with amyloid fibrils.

Then, similar SPR experiments carried out with a range of nanoparticles concentrations (from 0.02 up to 6.4 mM expressed in peptide concentrations) showed that both AGuIX@PEG@LPFFD@Cy5.5 (Fig. 5b1) and AGuIX@PEG@KLVFF@Cy5.5 (Fig. 5b2) interact with Aβ(1–42) fibrils. The sensorgrams obtained were fitted with the Biaevaluation^®^ software (Langmuir 1:1). Each experiment was repeated three times and led to the dissociations constants in the range of 534 ± 134 µM for AGuIX@PEG@KLVFF@Cy5.5 and of 261 ± 59 µM for AGuIX@PEG@LPFFD@Cy5.5. Although the K_d_ values are of several of hundred µM, these results demonstrate that the capacity of KLVFF and LPFFD to bind Aβ(1–42) fibrils is conserved after their grafting on AGuIX, with affinity of the same order of magnitude than those obtain with free peptides.

The specificity of these interactions for Aβ(1–42) fibrils was assessed in the same range of concentration of nanoparticles on a chip coated with V30M-TTR fibrils. As illustrated on Fig. 5c, 141 s after the end of nanoparticles injection, whereas both AGuIX@PEG@LPFFD@Cy5.5 and AGuIX@PEG@KLVFF@Cy5.5 displayed an interaction on Aβ(1–42) fibrils (blue curves) dependent on nanoparticle concentrations injected, no specific interaction of any functionalized nanoparticles was recorded on V30M-TTR fibrils (red curves). This confirms the discriminatory feature of AGuIX@PEG@LPFFD@Cy5.5 and AGuIX@PEG@KLVFF@Cy5.5 for Aβ(1-42) fibrils, supporting that the interactions between AGuIX@PEG@LPFFD@Cy5.5 and AGuIX@PEG@KLVFF@Cy5.5 with Aβ(1-42) fibrils are indeed mediated by the targeting peptides KLVFF and LPFFD.

Altogether, these in vitro experiments demonstrate that AGuIX nanoparticles functionalized with LPFFD or KLVFF, are able to bind and to discriminate Aβ(1–42) amyloid fibrils. Interestingly, it can be noticed that the grafting step of peptides on nanoparticles does not drastically modify the affinity of both peptide for the targeted fibrils.

### Vectorized-nanoparticles recognize Aβ plaques in AD mice brain

**Fig. 6:**
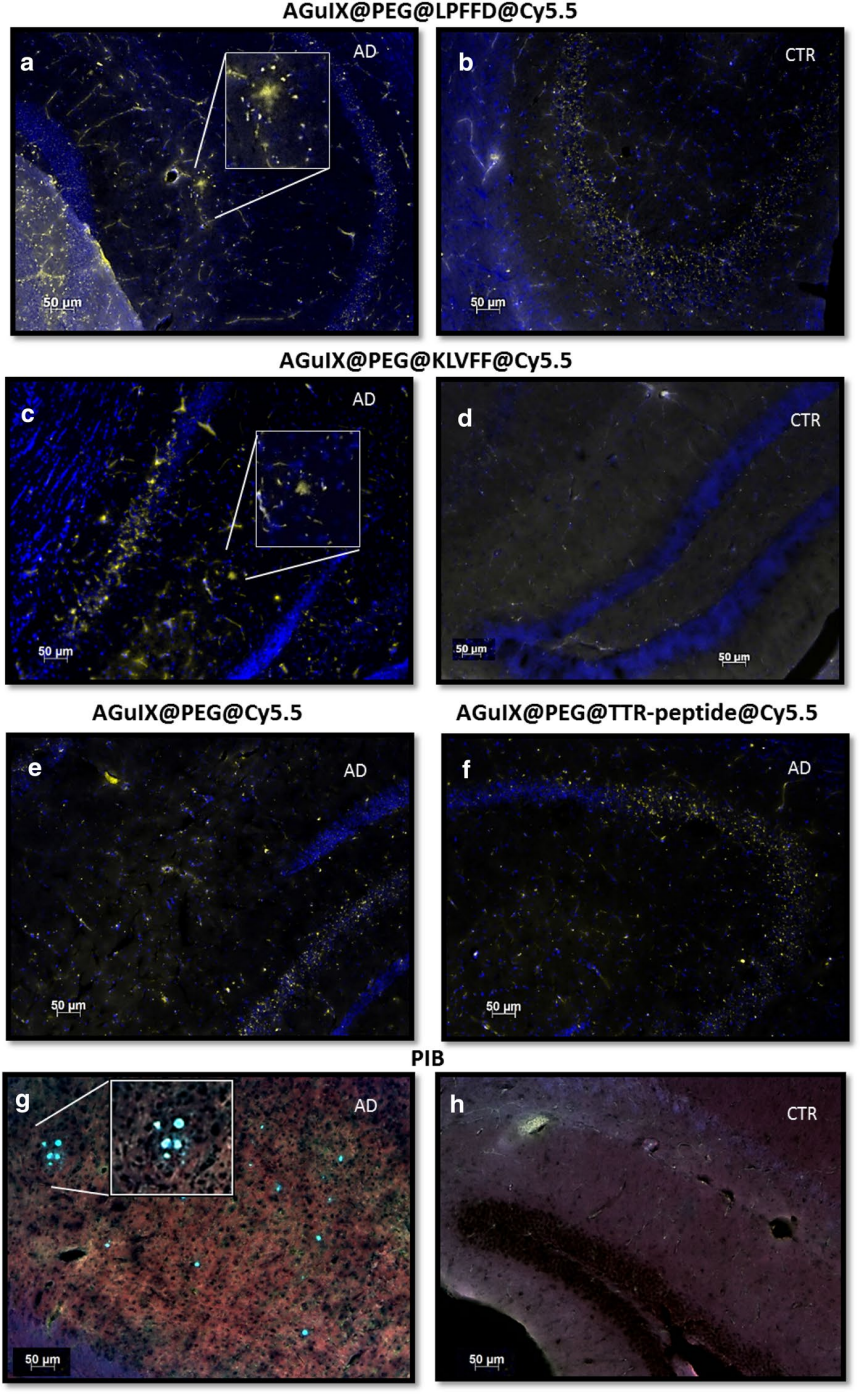
LPFFD and KLVFF vectorized nanoparticles hybridization on brain slices of APPswe/PS1A246/TTR transgenic (**a**, **c**, **e**, **f**, **g**) and control (**b**, **d**, **h**) mice. Imaging beta-amyloid plaques were visualized owing to Cy5.5 fluorescence grafted to AGuIX@PEG@LPFFD@Cy5.5 (zoom in **a**), and AGuIX@PEG@KLVFF@Cy5.5 (zoom in **c**), in the near-infra red (*yellow color*, λexc = 620 nm-λem = 642 nm). Cellular nucleus of brain sections were located using Hoechst dye staining (*blue color* λexc = 350 nm-λem = 405 nm). AGuIX@PEG@LPFFD@Cy5.5 and AGuIX@PEG@KLVFF@Cy5.5 binding specificity test were performed on brain section without amyloid plaques (**b**, **d**), and with nanoparticle without peptides, AGuIX@PEG@Cy5.5 (**e**), or with a non-related Aβ(1-42) peptide, AGuIX@PEG@TTR-peptide@Cy5.5 (**f**) on brain section with amyloid plaques. Positive control of amyloid burden was performed using PIB staining on brain sections with (**g**) and without amyloid plaques (**h**). Ligands binding are detected by fluorescence imaging of PIB at 400 nm (*blue color*, **g**). Tissue architecture was* highlighted* by imaging actin-phalloidin staining (**g**, **h**, *red color*, λexc = 540 nm-λem = 570 nm)

As next step, the ability of both AGuIX@PEG@LPFFD@Cy5.5 and AGuIX@PEG@KLVFF@Cy5.5 to detect Aβ amyloid plaques into the brain of Alzheimer’s disease animal model was tested. For this purpose, immunohistochemical experiments were performed on APPswe/PS1A246E/TTR(−/−) transgenic mouse brain sections. Such 10 months old transgenic mice display amyloid plaques in the cortex and the hippocampus [50]. Similar experiments were performed on control animals of APPswe/PS1A246E/TTR(±), which do not contain amyloid deposits in the cortex and the hippocampus (Fig. 6b, d). AGuIX@PEG@LPFFD@Cy5.5 or AGuIX@PEG@KLVFF@Cy5.5 was incubated on brain sections with or without amyloid deposits and visualized, owing to the grafted-Cy5.5 fluorescence by optic microscopy in the near infrared, while cellular nucleus were located using Hoechst dye.

As illustrated on Fig. 6, strong Cy5.5 fluorescence was visualized in AD brain sections, in specific structure (zoomed in a square Fig. 6a, c), with a core surrounded by diffuse fluorescence and by dots. These observations are characteristic of AD lesions constituted by amyloid plaque with cored and diffused deposits and dystrophic neurites in the vicinity of the plaque [51]. These structures are mainly constituted by Aβ(1–42) peptide into fibrillar β-sheet structure [52]. Similar experiments, on control brain sections without amyloid plaques, did not display such fluorescent staining as visualized in Fig. 6b and d.

The specificity of functionalized nanoparticles binding was verified, by incubating nanoparticles without grafted-peptides, AGuIX@PEG@Cy5.5, on brain section containing amyloid burden. As expected, no amyloid plaque was stained by Cy5.5 fluorescence in hippocampus region (Fig. 6e). Although, some yellow dots were noticeable on the vessels and in the CA3 cell layer of the hippocampus and control immunostaining experiments with Cy5.5 alone indicated this staining constitutes a background due to the Cy5.5 restricted to blood vessels (Additional file 1). Whereas, various other cyanine derivative dyes [53] can bind amyloid fibrils, the Cy5.5 molecule does not stain fibrils as observed in vitro on fibrils (personal data) and on brain section of AD mouse model [54].

The selectivity of AGuIX@PEG@LPFFD@Cy5.5 and AGuIX@PEG@KLVFF@Cy5.5 for Aβ(1–42) amyloid deposits was also assessed using a nanoparticle grafted with a peptide non related to Aβ(1–42) fibrils, AGuIX@PEG@TTR-peptide@Cy5.5. Immunohistochemistry performed on AD brain sections with this non related Aβ-peptide grafted nanoparticle, showed a low background related to cyanine5.5 fluorescence (Fig. 6f).

The Aβ burden of AD mouse model was evaluated using PIB for immunohistochemical staining as fluorescent ligand on AD brain. Figure 6g resulted from the merge of both staining and revealed robust fluorescent dots with diffusing labeling localized in AD mouse hippocampus (see in zoom square Fig. 6g). Such picture could be correlated with compact/cored amyloid plaque [55] and diffuse plaques [53].

Altogether, these results indicated that both AGuIX@PEG@LPFFD@Cy5.5 and AGuIX@PEG@KLVFF@Cy5.5, bind selectively amyloid plaque constituted by Aβ protein on ex vivo AD mouse hippoacampus.

## Conclusions

In this work, ultra-small gadolinium-nanoparticles were successfully grafted with two peptides targeting Aβ(1–42) fibrils (KLVFF and LPFFD). Although these peptides are described as β-breakers due to their ability to interact with Aβ monomer and to inhibit the formation of amyloid oligomers [56] and fibrils [36, 56, 57], they were shown to selectively interact with Aβ(1–42) amyloid fibrils as well. The binding affinity of the grafted peptide for Aβ(1–42) fibrils is in the range of the hundredth of µM, this remaining compatible with the injected dose usually used in clinical MRI applications and particularly for diagnosis. Histological examination of the nanoparticles proved their ability to preferentially interact with amyloid plaques present in the brain of AD mouse model confirming their targeting potential. The selective interaction of the functionalized nanoparticles with Aβ(1–42) fibrils was confirmed by the lack of affinity for V30M-TTR fibrils, amyloid fibrils implicated in FAP, another type of amyloidosis.

Characterization of these new nanoparticles confirms that they possess several of the features needed for biomedical neuroimaging: relaxivity’s measures showed that functionalized AGuIX conserve high positif MRI contrast allowing MRI signal enhancement, facilitating image interpretation [58]; the absence of AGuIX accumulation in certain organs [32], such as the liver or spleen, and their rapid renal elimination allow their consideration for in vivo injection, as it’s well known that such phenomena may constitute a limit to the use of certain contrast agents, as reported for iron oxide nanoparticles [59], where their long lasting presence can be associated with cellular cytotoxic effects [60, 61].

Besides the high MRI contrast of AGuIX, due to their enrichment in gadolinium chelates, these nanoparticles can be formulated to create a multimodal imaging platform, and/or by coupling ^68^Ga derivatives for PET detection [62] and for instance by covalently grafting a near infrared fluorophore for small animal. In conclusion, the ability of targeting and typing amyloid fibrils, by these new multifunctional nanoparticles, constitutes a first step of a promise and powerful strategy for a reliable early detection and identification of amyloidoses. Their future development will constitute a diagnostic and theranostic tool for the monitoring of the disease progression and the biochemical effects of drugs.

## Methods

### Chemicals

*N*-(3-Dimethylaminopropyl)-*N*′-ethylcarbodiimide hydrochloride (EDC, >98.0 %), N-hydroxysuccinimide (NHS, >97.0 %), gadolinium chloride hexahydrate ([GdCl_3_·6H_2_O], 99 %), sodium hydroxide (NaOH, 99.99 %), hydrochloric acid (HCl, 36.5–38 %), sodium chloride (NaCl, >99.5 %), dimethyl sulfoxide (DMSO, >99.5 %), acetonitrile (CH_3_CN, >99.9 %), trifluoroacetic acid (TFA, >99 %), trinitrobenzene sulfonic acid (TNBS) 1 M in water, sodium dodecyl sulfate (SDS, >99 %), (3-aminopropyl)triethoxysilane (APTES, >98 %), tetraethyl orthosilicate (TEOS; >98 %) and Poly(ethylene glycol) bis(carboxymethyl) ether (PEG) with an average molecular weight Mn = 250 g mol^−1^ were purchased from Aldrich Chemical (France) and used without further purification. Diethylene glycol (DEG, 99 %) was purchased from SDS Carlo Erba (France). Acetone (reagent grade) was purchased from Sodipro (France) and was used as received. Freeze-dried full peptides Aβ(1–42), small Aβ specific-peptide (LPFFD and KLVFF) (>98 %) and small TTR targeter peptide (>98 %) were purchased from Genecust (Luxembourg). Recombinant human V30M-TTR protein was obtained following cloning its gene in the plasmid pQE81L and its expression in *Escherichia coli* (M15). Briefly, bacterial cultures were performed in LB medium, 100 µg mL^−1^ ampicillin and 25 µg mL^−1^ kanamycin, at 37 °C, under constant orbital shaking. Protein production was pursued for 7 h after IPTG induction when the absorbance was 0.700 at 600 nm. The bacterial pellet of 50 mL of culture was resuspended in sodium phosphate 50 mM, pH 8, sodium chloride 300 mM, imidazole 10 mM, betamercaptoethanol 10 mM, supplemented with 500 µL of a protease inhibitor, phenylmethylsulfonyl fluoride (Sigma, #78830), at 1 mM final and a cocktail of protease inhibitor EDTA-free (Roche, #11873580001). The suspension was sonicated during a total time of 6 min with repeated cycles of 5/7 s off with an amplitude of 20 %, then centrifuged during 30 min at 100,000*g*, to pellet cellular debris. The supernatant was filtered on 0.2 µm cut off filters. The purification of the protein was performed on immobilized metal ion chromatography (Chelating Fast Flow, Amersham #17057501), and dialyzed against 20 mM·NH_4_HCO_3_ (membrane cutoff 1/10,000; 3500 MWCO Spectrapore). Finally, the protein was freeze-dried. The protein quality was checked by SDS PAGE gel 15 %. The Pittsburgh compound-B (PIB) derivative molecule was synthetized according to the procedure already described in the literature [63]. Starting from the p-anisidine, the p-nitrobenzoyl amide was readily obtained and then the corresponding thioamide was prepared by reaction with Lawesson’s reagent. Finally, an oxidative cyclization was done to lead to the benzothiazole derivative, and the nitro function was reduced to give compound (Synthesis’ details in Additional file 1).

### Nanoparticles synthesis and functionalization

The AGuIX Nps were provided by Nano-H SAS (Saint-Quentin Fallavier, France) and the DOTAGA anhydride (1,4,7,10-tetraazacyclododecane-1-glutaric anhydride-4-7-10-triacetic acid) was purchased from CheMatech (Dijon, France). AGuIX syntheses were performed at room temperature. Nanoparticles concentrations are stated in mol L^−1^ of Gd^3+^. AGuIX were synthetized as previously described [29] (see Additional file 1). These Nps are composed of a polysiloxane network which is surrounded by covalently grafted DOTAGa(Gd^3+^) chelate. During the first step, Poly(ethylene glycol) bis(carboxymethyl) ether (PEG) was grafted on AGuIX. The freeze-dried AGuIX nanoparticles were dispersed in water to reach a concentration of Gd^3+^ of 500 mM and then diluted ten times with a solution of diethylene glycol (DEG) at 80 °C. In parallel, the carboxylic acid functions of the PEG chains were activated with EDC and NHS coupling reagents (EDC/NHS/PEG molar ratio 10:20:1). The NHS-ester formation was performed in anhydrous DMSO at 100 mM in PEG under stirring at room temperature for 30 min. Then, the NHS-ester PEG solution was added to the nanoparticles suspension (PEG/Gd molar ratio 3:1) and the reaction mixture left for 12 h at room temperature. Finally, the Nps were diluted in ultrapure water in order to reach a volume percentage (%v) of DMSO below 5 %. Purification of the nanoparticles was performed by tangential flow filtration on a 5 kDa molecular cutoff membrane. This step allows removing the unreacted reagents and the degraded nanoparticles. The AGuIX@PEG nanoparticles were freeze-dried using a Christ Alpha 1–2 lyophilizer until the covalent conjugation of the Aβ amyloid peptides. In a second time, freeze-dried AGuIX@PEG nanoparticles were first dispersed in water to reach a concentration of Gd^3+^ of 500 mM, and then successively diluted ten times with DEG at 80 °C and then two times with anhydrous DMSO, leading to final concentration of Gd^3+^ of 25 mM. The carboxylic functions of the NPs were activated by EDC and NHS (EDC/NHS/Gd molar ratio 10:20:1) under stirring at room temperature for 30 min. The activated Nps suspension was divided into two aliquots: one to be grafted with each of the two Aβ specific peptides. Each peptide, dissolved at 100 mg mL^−1^ in anhydrous DMSO, was added to the Nps suspension and stirred for 12 h at room temperature. The nanoparticles were then diluted in ultrapure water in order to reach a concentration in DMSO below 5 %v and purified by tangential filtration on a 5 kDa molecular cutoff membrane. This step allows removing the EDC/NHS activators, the unreacted peptides and the degraded nanoparticles. The suspension of purified AGuIX@PEG@Peptide nanoparticles was set at 100 mM of Gd^3+^ in ultrapure water and the pH was adjusted to 7. Finally, AGuIX@PEG@Peptide Nps were grafted with the fluorophore, Cyanine 5.5 NHS, which was dissolved in anhydrous DMSO at 2.5 mg mL^−1^. This mixture was slowly added to the nanoparticles suspension (Cy5.5/Gd molar ratio 1:1000). After 4 h of reaction, the mixture was purified by tangential filtration with a Vivaspin^®^ tube with a cutoff of 5 kDa in order to remove the ungrafted cyanine molecules, the NHS formed and the degraded particles. Finally the nanoparticles AGuIX@PEG@Peptide@Cy5.5 were freeze-dried for storage using a Christ Alpha 1–2 lyophilizer. They are stable for months without alteration at room temperature.

### Relaxivity measurements

Relaxivity measurements were performed on a Bruker^®^ minispec mq60NMR analyzer (Brucker, USA) at 37 °C at 1.4 T (60 MHz). Samples were measured at a specific Gd^3+^ concentration (mM), calculated from ICP-OES. The longitudinal relaxation time T_1_ and the transverse relaxation time T_2_ (s) were measured at 60 MHz and at 37 °C. Then the relaxivities r_i_ (s^−1^ mM^−1^) were obtained according to the following formula:

$$ \left( {\frac{1}{{T_{i} }}} \right)measured = \left( {\frac{1}{{T_{i} }}} \right)water + r_{i} \left[ {Gd^{3 + } } \right] $$

where i = 1 or 2.

### Dynamic light scattering (DLS)

Hydrodynamic diameters (D_H_) of the Nps were determined with a Zetasizer NanoS (laser He–Ne 532 nm) from Malvern Instrument^®^ (5 mW, with 173° NIBS detector and narrow band filter). Attenuator was optimized by the device and position was set to the center of the cell. To perform the measurement, 1 mL of nanoparticles suspensions at 10 mM Gd^3+^ was poured in a 12 mm square polystyrene disposable cuvette after filtration on a 0.2 µm cut-off nylon filter. Each measurement was repeated 8 times.

### High performance liquid chromatography (HPLC)

Gradient HPLC analysis was carried out using the Shimadzu^®^ Prominence series UFLC system equipped with a CBM-20A controller bus module, an LC-20AD liquid chromatograph, a CTO-20A column oven, a SPD-20A UV–Visible detector allowing absorption measurement at two chosen wavelengths and a RF-20A fluorescence detector allowing fluorescence measurements at specific excitation and emission wavelengths. For all measurements, the UV–visible detectors were set respectively at 295 and 350 nm. Sample aliquots of 20 µL were injected in a 95 % solvent A—5 % solvent B (A = Milli-Q water/TFA 99.9:0.1 v/v; B = CH_3_CN/Milli-Q water/TFA 90:9.9:0.1 v/v/v) into a Jupiter C4 column (150 × 4.60 mm, 5 µm, 300 Å, Phenomenex^®^) at a flow rate of 1 mL min^−1^ over 5 min. In a second step, samples were eluted by a gradient from 5 to 90 % of solvent B in solvent A over 15 min. Finally, the concentration of solvent B was maintained over 5 min. Then, the concentration of solvent B was decreased to 5 % over a period of 5 min to re-equilibrate the system, followed by additional 5 min at this final concentration. Before each sample measurement, a baseline was recorded following the same conditions by loading Milli-Q water onto the injection loop.

### Inductively coupled plasma-optical emission spectroscopy (icp-oes) analysis

Determination of the gadolinium content in a sample was performed by ICP-OES analysis with a Varian 710-ES spectrometer. Before measuring Gd^3+^ concentration, samples of colloidal solution were dissolved in HNO_3_ 67 % at 80 °C for 2 h. The samples were then diluted 10-fold in HNO_3_ 5 %. Chemical analysis were also performed on the as-prepared samples at the Service Central d’Analyses du CNRS (Villeurbanne, France) by ICP-MS and enabled determining the C, N, Si contents with a precision of 0.5 %.

### Circular dichroism

Circular dichroism was used in order to determine the number of Aβ peptide grafted on the nanoparticles. Standard curves of peptide KLVFF and LPFFD are obtained by successive dilutions in distilled water (300 µL), respectively at 25, 50, 100 200 mg L^−1^ and 20, 50, 100, 200, 300 mg L^−1^. The freeze-dried nanoparticles were dissolved in distilled water at 5 mM, and diluted in 300 µL of distilled water at 0.03, 0.05 and 0.1 mM. Spectra of each sample were recorded in a 1 mm path length quartz cell on a JASCO J-815 CD spectrometer using a response time of 1 s, a scan speed of 200 nm min^−1^ and a bandwith of 4 nm. Thirty spectra were accumulated and averaged in far UV (200–250 nm) at 20 °C and quantification was done at 212 nm (KLVFF) and 219 nm (LPFFD). These wavelengths were selected on each peptide spectrum as the more characteristic point obtained in function of their concentrations. The molar ellipticity reported as a function of the peptide concentration and allowed determining the number of peptide per nanoparticles.

### Beta-amyloid fibrils growth

Freeze-dried Aβ(1–42) peptide (Genecust) was dissolved in distilled water at pH 11.0. The solution was filtrated through a spin 0.2 µm membrane filter (Millipore, #146560) to remove any aggregated species. The solution was stored at −20 °C. Aβ(1–42) fibrils were prepared by incubating monomer Aβ(1–42) peptide solution (final concentration at 100 μM) in Tyrode’s buffer (150 mM NaCl, 3 mM KCl, 10 mM HEPES pH 7.4, 10 mM glucose; pH 6.5) at 37 °C in 500 µL reaction volume on a rotating shaker (300 rpm) for 72 h. Fibril morphology formation was confirmed by TEM analysis (JEOL 1200EX).

### V30M-TTR amyloid fibrils growth

The protein V30M-TTR was directly dissolved in buffer A (KH_2_PO_4_ 10 mM, KCl 100 mM, EDTA 1 mM, adjusted to pH 7 and filtrated with a spin 0.2 µm) and buffer B (C_2_H_3_NaO_2_ 200 mM, KCl 100 mM, EDTA 1 mM, adjusted to pH 4.4, filtrated with a spin 0.2 µm), with the same volume of each buffer to obtain a final concentration at 32.3 µM of V30M-TTR protein (pH 4.6) in a 1 mL reaction volume. It was incubated on a rotating shaker (300 rpm) during 10 days at 70 °C. Fibril morphology formation was confirmed by TEM analysis.

### Transmission electronic microscopy

10 µL of Aβ(1–42) or V30M-TTR fibrils samples were adsorbed on to the clean face of a carbon film on mica sheet (carbon/mica interface) and negatively stained with 1 % (w/v) uranyl acetate. Micrographs were recorded at 80 kV using a JEOL 1200EX equipped with a Veleta camera (Olympus) and iTEM software or at 200 kV on a FEI Tecnai™ G2 Sphera.

### Binding assays

Twenty µM of Aβ(1–42) or V30M-TTR fibrils were incubated with PIB at 0.01, 0.1, 0.4, 0.8, 1.2, 2 and 4 µM, or 2 µM of AGuIX@PEG@KLVVF@Cy5.5 or AGuIX@PEG@LPFFD@Cy5.5, saturated with 3 mM AGuIX, for 60 min at room temperature in 500 µL of each prepared fibril buffer. A first measure of fluorescence was carried out on the mixture, which was then centrifuged for 25 min at 14000 rpm and 25 °C to obtain a pellet (containing fibrils) and a supernatant (containing free nanoparticles or PIB). The pellet was dispersed in 500 µL of fibril buffer. A second measure of fluorescence was realized both on the supernatant and the re-suspended pellet. According to the fluorescence properties of PIB (λ_exc_ = 350 nm, λ_em_ = 420 nm) or cyanine 5.5 (λ_exc_ = 600 nm, λ_em_ = 655 nm) spectrum of each sample was recorded in a 1 cm path length quartz cell on a CD-spectrophotometer (JASCO J-815) using a response time of 1 s, a scan speed of 100 nm min^−1^, an excitation bandwith of 5 nm and an emission bandwith of 10 nm. Three spectra were accumulated at 25 °C and averaged. Spectra are treated by subtracting of the spectrum recorded for the buffer. Michaelis binding model was used to determine the dissociation constant (K_D_) following the equation:

$$ {\text{y}} = {\text{Ym}}/\left( { 1 + \left( {{\text{Kd}}/{\text{x}}} \right)} \right), $$

where Ym is the maximum of fluorescence (A.U).

### Surface plasmon resonance assays

The surface plasmon resonance assays were performed using a BIAcore^®^ 2000 biosensor. The instrument was equipped with a CM3 sensor chip (GE Healthcare^®^, Uppsala, Sweden). The interaction studies took place on a gold chip functionalized with carboxymethylated dextran. The sensor surface itself form one wall of a flow cell, which is an integral part of the microfluidic system. All SPR experiments were performed at 25 °C. HEPES buffer (HBS-P, GE Healthcare^®^, 0.01 M HEPES, 0.15 M NaCl, 0.005 % surfactant p20) was used as running buffer.

For immobilization, amine coupling reagents were obtained from the GE Healthcare^®^ kit containing 0.2 M EDC in water, 0.05 M NHS in water and ethanolamine-HCl pH 8.5. Prior immobilization, the size of the Aβ(1–42) fibrils was reduced using an ultrasonic disruptor Sonoplus HD 2070 (Banderlin, Germany). The sonication was performed on ice at 40 W during 20 s (pulse of 0.6 s; interval of 0.4 s; mode PULS, cycle 6) [64]. Aβ(1–42) and V30M-TTR fibrils were immobilized on the flow cell 2 and 4 respectively of a CM3 sensor chip with an amine coupling reaction. After the chip activation by EDC/NHS during 7 min, the fibrils were injected at: 10 µM in 10 mM sodium acetate pH 4 at a flow rate of 5 µL min^−1^ during 20 min for Aβ(1–42) fibrils or at 0.36 µM in 10 mM sodium acetate pH 5.5 at a flow rate of 5 µL min^−1^ during 100 min for V30M-TTR fibrils. The immobilization levels were around 1200 respond units (R.U.) for the Aβ(1–42) fibrils and 800 R.U. for the V30M-TTR fibrils. The flow cell 1 and 3 from this chip were activated by EDC/NHS during 7 min and then deactivated by ethanolamine during 7 min and used as reference surface.

Biacore^®^ experiments gathered data from 3 cycles for each studied analyte. One cycle consisted in the injection of the compound to be tested for 3 min. A waiting period of 2.5 min under running buffer (HBS-P) followed this step. Then the surface was regenerated, using the GE Healthcare^®^ regeneration scouting kit, to remove the analyte from the chip by injection of 2 M NaCl and 5 mM NaOH during 60 s. A stabilization period of 2 min under running buffer finished the cycle. For the kinetic assays, the nanoparticles were injected at the concentration of 0.02; 0.2; 0.4; 0.8; 1.6; 3.2 and 6.4 mM in peptide (corresponding to 0.1; 1; 2; 4; 8; 16 and 32 mM in Gd^3+^) in running buffer (HBS-P) with 10 % liquid dextran (non specific binding reducer, GE Healthcare^®^) to reduce unspecific interaction. The free peptides were injected at concentrations ranging from 44 to 704 µM. Each experiment began with five cycles injecting running buffer instead of analyte to stabilize the baseline of the apparatus. On the other hand, channel 1 or 3 were maintained in their carboxymethylated dextran native form to serve as a reference and observe possible non-specific interactions. Results of the interaction between the analytes and the Aβ(1–42) or V30M-TTR fibrils were represented on a sensorgram expressed as respectively FC2-1 or FC4-3 (RU-RUdextran) versus time. The data were processed by Biaevaluation software (Biacore) and fitted using the 1:1 Langmuir binding model to obtain the equilibrium dissociation constant (K_d_). The step was performed for each of the three sets of experiments. The value of R.U. obtained 141 s after the end of the injection was compared for the AGuIX@PEG, AGuIX@PEG@Cy5.5, AGuIX@PEG@LPFFD@Cy5.5 and AGuIX@PEG@KLVFF@Cy5.5 at 2.5 mM in Gd^3+^ on Aβ(1–42) fibrils and V30M-TTR fibrils.

### Animal models

APPswe/PS1A246E/TTR mouse model was generated in JM Saraiva’s lab by crossing APPswe/PS1A246E mice (B6/C3H background) with TTRnull mice (TTR −/−) (SV129 background) [65]. These transgenic mice co-express a chimeric mouse-human amyloid-protein precursor (APP) bearing a human A domain with mutations (K595N and M596L) linked to Swedish (Swe) familial AD (FAD) pedigrees and human presenilin 1 (PS1) bearing a mutation (A246E) which also causes FAD. Expression of both transgenes is under the control of the mouse prion protein promoter (PrP). Animals used in this work were female APP/PSEN/TTR(−/−) 10 months old, displaying a dramatic amyloid plaques burden. Six months old male APP/PSEN/TTR(±), displaying rare plaques and very low levels of detergent- and formic acid-soluble Aβ40 and Aβ42 proteins in the transgenic male brain [66] were used as control. Animals were housed in a controlled environment (12-h light/dark cycle; temperature, 22 ± 2 °C; humidity, 45–65 %), with food and water freely available. All procedures involving animals were carried out in accordance with the European Communities Council Directive.

### Immunohistochemistry and microscopy

Mice were deeply anesthetized with a mixture of ketamine and medetomidine. Brains were removed from the skull and bisected longitudinally: each half was fixed for 24 h at 4 °C in 10 % neutral buffered formalin and then transferred to a 30 % sucrose solution for cryoprotection before cryostat sectioning (10 µm) for immunohistochemical analyses. Sections were mounted on glass slides and allowed to dry at room temperature for a few minutes. They were washed three times in phosphate buffer saline (PBS), and incubated into permeabilization-saturation buffer [PBS 1X, 0.3 % triton, 1 % bovine serum albumin] for 1 h at RT. Then incubation with, AGuIX@PEG@KLVVF@Cy5.5, AGuIX@PEG@LPFFD@Cy5.5, AGuIX@Cy5.5 or AGuIX@PEG@TTR@Cy5.5 nanoparticles at 3 nM of Cy5.5 (corresponding to 2 mM Gd^3+^) with, for non-specific saturation, 3 mM ungrafted nanoparticles, or PIB at 1 µM were incubated on section in the same buffer for 1h30 at RT. For amyloid burden control, sections were directly stained with Thioflavin-S (at 0.05 % in EtOH 50 %, Sigma, #T1892) according to standard practice. Cell staining was performed using the phalloidin-TRITC (Sigma, #P1951) in the case of slices treated with PIB. After the PBS wash, all the other sections were coverslipped with dabco-mowiol mounting medium with additional Hoechst to stain cell nucleus. Fluorescent images were observed using the proper filter for each marker: cyanine 5.5: λ_excitation_ = 620 nm-λ_emission_ = 642 nm, PIB or Hoechst: λ_excitation_ = 350 nm-λ_emission_ = 405 nm, phalloidin: λ_excitation_ = 540 nm-λ_emission_ = 570 nm and Thioflavin-S: λ_excitation_ = 450 nm-λ_emission_ = 488 nm using a Zeiss microscope Axiovert 200 M. Images were analyzed using Carl Zeiss AxioVision software.

## Additional file

10.1186/s12951-016-0212-y Characterization of functionalized nanoparticles. **Figure S2.** Zeta potential versus pH of AGuIX (black) and AGuIX@PEG (red). **Figure S3.** Reaction scheme for the synthesis of AGuIX@PEG@Peptide@Cy5.5. **Figure S4.** Chromatograms obtained with at UV-Visible absorption at λ =295 nm of the AGuIX@PEG, AGuIX@PEG@LPFFD and AGuIX@PEG@KLVFF . Elementary Analyses of grafted nanoparticles: **Table S1.** Molar ratio deduced from experimental weight percentage; **Table S2.** Elementary analyses given in weight percent of element in the compound; **Table S3.** Comparison of the primary amine content evaluated by elementary analysis and TNBS assays. These results allow assessing the molecular formula obtained by elementary analysis. **Figure S5.** Standard curves of amine quantification in APTES/TEOS equimolar mixture by TNBS assays. **Figure S6.** The cyanine 5.5 presents characteristic absorption and emission wavelength λexc max Cy5.5 = 675 nm and λem max Cy5.5 = 692 nm. **Table S4.** The yield of the grafting was calculated thanks to the comparison of the cyanine fluorescence and the DOTA(Gd^3+^) chelate before and after removal of the ungrafted dye by tangential purification. **Figure S7.** Effect of nanoparticles on cell viability. **Figure S8.** PIB derivative synthesis details. **Figure S9.** Control of the non-binding of Cy5.5 dye to amyloid plaque in brain section of AD mouse model.

## Abbreviations

Aβ: amyloid-beta protein
AD: Alzheimer’s disease
FAP: familial amyloid polyneuropathy
TTR: transthyretin protein
V30M-TTR: mutated-(V30M) transthyretin protein
MRI: magnetic resonance imaging
PET: positron emission tomography
SPECT: single photon emission CT
PIB: Pittsburgh compound B
Cy5.5: cyanine 5.5
APTES: (3-Amino)propyltriethoxy silane
PEG: polyethylene glycol diacid
FTIR: Fourier Transform Infrared spectroscopy
MTT: 3-(4,5-dimethylthiazol-2-yl)-2,5-diphenyltetrazolium bromide
SPR: surface plasmon resonance
R.U.: resonance Unit

## References

[1] Knowles TP, Vendruscolo M, Dobson CM (2014). The amyloid state and its association with protein misfolding diseases. Nat Rev Mol Cell Biol.

[2] Chiti F, Dobson CM (2006). Protein misfolding, functional amyloid, and human disease. Annu Rev Biochem.

[3] Ueda M, Ando Y (2014). Recent advances in transthyretin amyloidosis therapy. Transl Neurodegener.

[4] Perrin RJ, Fagan AM, Holtzman DM (2009). Multimodal techniques for diagnosis and prognosis of Alzheimer’s disease. Nature.

[5] Dementia and risk reduction: an analysis of protective and modifiable factors. World Alzheimer Report 2014.

[6] Ferreira IL, Resende R, Ferreiro E, Rego AC, Pereira CF (2010). Multiple defects in energy metabolism in Alzheimer’s disease. Curr Drug Targets.

[7] Hsia AY, Masliah E, McConlogue L, Yu GQ, Tatsuno G, Hu K, Kholodenko D, Malenka RC, Nicoll RA, Mucke L (1999). Plaque-independent disruption of neural circuits in Alzheimer’s disease mouse models. Proc Natl Acad Sci USA.

[8] Chapman PF, White GL, Jones MW, Cooper-Blacketer D, Marshall VJ, Irizarry M, Younkin L, Good MA, Bliss TV, Hyman BT (1999). Impaired synaptic plasticity and learning in aged amyloid precursor protein transgenic mice. Nat Neurosci.

[9] Walsh DM, Klyubin I, Fadeeva JV, Cullen WK, Anwyl R, Wolfe MS, Rowan MJ, Selkoe DJ (2002). Naturally secreted oligomers of amyloid beta protein potently inhibit hippocampal long-term potentiation in vivo. Nature.

[10] Hardy JA, Higgins GA (1992). Alzheimer’s disease: the amyloid cascade hypothesis. Science.

[11] Selkoe DJ (1991). The molecular pathology of Alzheimer’s disease. Neuron.

[12] Braak H, Braak E (1997). Frequency of stages of Alzheimer-related lesions in different age categories. Neurobiol Aging.

[13] Thal DR, Rub U, Orantes M, Braak H (2002). Phases of A beta-deposition in the human brain and its relevance for the development of AD. Neurology.

[14] Frisoni GB, Laakso MP, Beltramello A, Geroldi C, Bianchetti A, Soininen H, Trabucchi M (1999). Hippocampal and entorhinal cortex atrophy in frontotemporal dementia and Alzheimer’s disease. Neurology.

[15] McKhann G, Drachman D, Folstein M, Katzman R, Price D, Stadlan EM (1984). Clinical diagnosis of Alzheimer’s disease: report of the NINCDS-ADRDA Work Group under the auspices of department of health and human services task force on Alzheimer’s disease. Neurology.

[16] Nordberg A (2010). Amyloid imaging in early detection of Alzheimer’s disease. Neurodegener Dis.

[17] Coimbra A, Williams DS, Hostetler ED (2006). The role of MRI and PET/SPECT in Alzheimer’s disease. Curr Top Med Chem.

[18] Mosconi L, Berti V, Glodzik L, Pupi A, De Santi S, de Leon MJ (2010). Pre-clinical detection of Alzheimer’s disease using FDG-PET, with or without amyloid imaging. J Alzheimers Dis.

[19] Mistur R, Mosconi L, Santi SD, Guzman M, Li Y, Tsui W, de Leon MJ (2009). Current challenges for the early detection of Alzheimer’s disease: brain imaging and CSF studies. J Clin Neurol.

[20] Rabinovici GD, Jagust WJ (2009). Amyloid imaging in aging and dementia: testing the amyloid hypothesis in vivo. Behav Neurol.

[21] Hellstrom-Lindahl E, Westermark P, Antoni G, Estrada S (2014). In vitro binding of [(3)H]PIB to human amyloid deposits of different types. Amyloid.

[22] Kadir A, Marutle A, Gonzalez D, Scholl M, Almkvist O, Mousavi M, Mustafiz T, Darreh-Shori T, Nennesmo I, Nordberg A (2011). Positron emission tomography imaging and clinical progression in relation to molecular pathology in the first Pittsburgh compound B positron emission tomography patient with Alzheimer’s disease. Brain.

[23] Kantarci K, Yang C, Schneider JA, Senjem ML, Reyes DA, Lowe VJ, Barnes LL, Aggarwal NT, Bennett DA, Smith GE (2012). Antemortem amyloid imaging and beta-amyloid pathology in a case with dementia with Lewy bodies. Neurobiol Aging.

[24] Bao G, Mitragotri S, Tong S (2013). Multifunctional nanoparticles for drug delivery and molecular imaging. Annu Rev Biomed Eng.

[25] Gao N, Sun H, Dong K, Ren J, Qu X (2015). Gold-nanoparticle-based multifunctional amyloid-beta inhibitor against Alzheimer’s disease. Chemistry.

[26] Larbanoix L, Burtea C, Laurent S, Van Leuven F, Toubeau G, Vander Elst L, Muller RN (2010). Potential amyloid plaque-specific peptides for the diagnosis of Alzheimer’s disease. Neurobiol Aging.

[27] Lux F, Mignot A, Mowat P, Louis C, Dufort S, Bernhard C, Denat F, Boschetti F, Brunet C, Antoine R (2011). Ultrasmall rigid particles as multimodal probes for medical applications. Angew Chem Int Ed Engl.

[28] Mignot A, Truillet C, Lux F, Sancey L, Louis C, Denat F, Boschetti F, Bocher L, Gloter A, Stephan O (2013). A top-down synthesis route to ultrasmall multifunctional Gd-based silica nanoparticles for theranostic applications. Chemistry.

[29] Le Duc G, Roux S, Paruta-Tuarez A, Dufort S, Brauer E, Marais A, Truillet C, Sancey L, Perriat P, Lux F, Tillement O (2014). Advantages of gadolinium based ultrasmall nanoparticles vs molecular gadolinium chelates for radiotherapy guided by MRI for glioma treatment. Cancer Nanotechnol.

[30] Bianchi A, Moncelet D, Lux F, Plissonneau M, Rizzitelli S, Ribot EJ, Tassali N, Bouchaud V, Tillement O, Voisin P, Cremillieux Y (2015). Orotracheal administration of contrast agents: a new protocol for brain tumor targeting. NMR Biomed.

[31] Morlieras J, Chezal JM, Miot-Noirault E, Vidal A, Besse S, Kryza D, Truillet C, Mignot A, Antoine R, Dugourd P (2013). In vivo evidence of the targeting of cartilaginous tissue by pyridinium functionalized nanoparticles. Chem Commun (Camb).

[32] Sancey L, Kotb S, Truillet C, Appaix F, Marais A, Thomas E, van der Sanden B, Klein JP, Laurent B, Cottier M (2015). Long-term in vivo clearance of gadolinium-based AGuIX nanoparticles and their biocompatibility after systemic injection. ACS Nano..

[33] Lowe TL, Strzelec A, Kiessling LL, Murphy RM (2001). Structure-function relationships for inhibitors of beta-amyloid toxicity containing the recognition sequence KLVFF. Biochemistry.

[34] Hilbich C, Kisters-Woike B, Reed J, Masters CL, Beyreuther K (1992). Substitutions of hydrophobic amino acids reduce the amyloidogenicity of Alzheimer’s disease beta A4 peptides. J Mol Biol.

[35] Soto C, Kindy MS, Baumann M, Frangione B (1996). Inhibition of Alzheimer’s amyloidosis by peptides that prevent beta-sheet conformation. Biochem Biophys Res Commun.

[36] Soto C, Sigurdsson EM, Morelli L, Kumar RA, Castano EM, Frangione B (1998). Beta-sheet breaker peptides inhibit fibrillogenesis in a rat brain model of amyloidosis: implications for Alzheimer’s therapy. Nat Med.

[37] Tjernberg LO, Naslund J, Lindqvist F, Johansson J, Karlstrom AR, Thyberg J, Terenius L, Nordstedt C (1996). Arrest of beta-amyloid fibril formation by a pentapeptide ligand. J Biol Chem.

[38] Tojo K, Sekijima Y, Kelly JW, Ikeda S (2006). Diflunisal stabilizes familial amyloid polyneuropathy-associated transthyretin variant tetramers in serum against dissociation required for amyloidogenesis. Neurosci Res.

[39] Lashuel HA, Lai Z, Kelly JW (1998). Characterization of the transthyretin acid denaturation pathways by analytical ultracentrifugation: implications for wild-type, V30M, and L55P amyloid fibril formation. Biochemistry.

[40] Ikeda S, Nakazato M, Ando Y, Sobue G (2002). Familial transthyretin-type amyloid polyneuropathy in Japan: clinical and genetic heterogeneity. Neurology.

[41] Sashidhar RB, Capoor AK, Ramana D (1994). Quantitation of epsilon-amino group using amino acids as reference standards by trinitrobenzene sulfonic acid. A simple spectrophotometric method for the estimation of hapten to carrier protein ratio. J Immunol Methods.

[42] Helm L, Merbach A, Toth E (2013). The chemistry of contrast agents in medical magnetic resonance imaging.

[43] Lauffer RB (1987). Paramagnetic metal-complexes as water proton relaxation agents for Nmr imaging—theory and design. Chem Rev.

[44] Xiao N, Gu W, Wang H, Deng YL, Shi X, Ye L (2014). T-1-T-2 dual-modal MRI of brain gliomas using PEGylated Gd-doped iron oxide nanoparticles. J Colloid Interface Sci.

[45] Xiong N, Dong XY, Zheng J, Liu FF, Sun Y (2015). Design of LVFFARK and LVFFARK- functionalized nanoparticles for inhibiting amyloid beta-protein fibrillation and cytotoxicity. ACS Appl Mater Interfaces.

[46] Fotakis G, Timbrell JA (2006). In vitro cytotoxicity assays: comparison of LDH, neutral red, MTT and protein assay in hepatoma cell lines following exposure to cadmium chloride. Toxicol Lett.

[47] Sancey L, Lux F, Kotb S, Roux S, Dufort S, Bianchi A, Cremillieux Y, Fries P, Coll JL, Rodriguez-Lafrasse C (2014). The use of theranostic gadolinium-based nanoprobes to improve radiotherapy efficacy. Br J Radiol.

[48] Miladi I, Le Duc G, Kryza D, Berniard A, Mowat P, Roux S, Taleb J, Bonazza P, Perriat P, Lux F (2013). Biodistribution of ultra small gadolinium-based nanoparticles as theranostic agent: application to brain tumors. J Biomater Appl.

[49] Liu J, Wang W, Zhang Q, Zhang S, Yuan Z (2014). Study on the efficiency and interaction mechanism of a decapeptide inhibitor of beta-amyloid aggregation. Biomacromolecules.

[50] Oliveira SM, Ribeiro CA, Cardoso I, Saraiva MJ (2011). Gender-dependent transthyretin modulation of brain amyloid-beta levels: evidence from a mouse model of Alzheimer’s disease. J Alzheimers Dis.

[51] Duyckaerts C, Delatour B, Potier MC (2009). Classification and basic pathology of Alzheimer disease. Acta Neuropathol.

[52] Glabe C (2001). Intracellular mechanisms of amyloid accumulation and pathogenesis in Alzheimer’s disease. J Mol Neurosci.

[53] Volkova KD, Kovalska VB, Balanda AO, Vermeij RJ, Subramaniam V, Slominskii YL, Yarmoluk SM (2007). Cyanine dye-protein interactions: looking for fluorescent probes for amyloid structures. J Biochem Biophys Methods.

[54] Medarova Z, Bonner-Weir S, Lipes M, Moore A (2005). Imaging beta-cell death with a near- infrared probe. Diabetes.

[55] Ikonomovic MD, Klunk WE, Abrahamson EE, Mathis CA, Price JC, Tsopelas ND, Lopresti BJ, Ziolko S, Bi W, Paljug WR (2008). Post-mortem correlates of in vivo PiB-PET amyloid imaging in a typical case of Alzheimer’s disease. Brain.

[56] Lockhart A, Lamb JR, Osredkar T, Sue LI, Joyce JN, Ye L, Libri V, Leppert D, Beach TG (2007). PIB is a non-specific imaging marker of amyloid-beta (Abeta) peptide-related cerebral amyloidosis. Brain.

[57] Viet MH, Ngo ST, Lam NS, Li MS (2011). Inhibition of aggregation of amyloid peptides by beta- sheet breaker peptides and their binding affinity. J Phys Chem B.

[58] Wengenack TM, Jack CR, Garwood M, Poduslo JF (2008). MR Microimaging of amyloid plaques in Alzheimer’s disease transgenic mice. Eur J Nucl Med Mol Imaging.

[59] Austen BM, Paleologou KE, Ali SA, Qureshi MM, Allsop D, El-Agnaf OM (2008). Designing peptide inhibitors for oligomerization and toxicity of Alzheimer’s beta-amyloid peptide. Biochemistry.

[60] Elias A, Tsourkas A (2009). Imaging circulating cells and lymphoid tissues with iron oxide nanoparticles. Hematol Am Soc Hematol Educ Program..

[61] Singh N, Jenkins GJ, Asadi R, Doak SH. Potential toxicity of superparamagnetic iron oxide nanoparticles (SPION). Nano Rev. 2010; 1. 10.3402/nano.v1i0.5358 PMC3215220 22110864

[62] Truillet C, Bouziotis P, Tsoukalas C, Brugiere J, Martini M, Sancey L, Brichart T, Denat F, Boschetti F, Darbost U (2015). Ultrasmall particles for Gd-MRI and (68) Ga-PET dual imaging. Contrast Media Mol Imaging.

[63] Martins AF, Morfin JF, Kubickova A, Kubicek V, Buron F, Suzenet F, Salerno M, Lazar AN, Duyckaerts C, Arlicot N (2013). PiB-conjugated, metal-based imaging probes: multimodal approaches for the visualization of beta-amyloid plaques. ACS Med Chem Lett.

[64] Hasegawa K, Ono K, Yamada M, Naiki H (2002). Kinetic modeling and determination of reaction constants of Alzheimer’s beta-amyloid fibril extension and dissociation using surface plasmon resonance. Biochemistry.

[65] Episkopou V, Maeda S, Nishiguchi S, Shimada K, Gaitanaris GA, Gottesman ME, Robertson EJ (1993). Disruption of the transthyretin gene results in mice with depressed levels of plasma retinol and thyroid hormone. Proc Natl Acad Sci USA.

[66] Borchelt DR, Ratovitski T, van Lare J, Lee MK, Gonzales V, Jenkins NA, Copeland NG, Price DL, Sisodia SS (1997). Accelerated amyloid deposition in the brains of transgenic mice coexpressing mutant presenilin 1 and amyloid precursor proteins. Neuron.

