# Supplementary material for: Gd-nanoparticles functionalization with specific peptides for ß-amyloid plaques targeting
Source: J Nanobiotechnology. 2016 Jul 25;14:60. doi: 10.1186/s12951-016-0212-y (PMC4960888; doi:10.1186/s12951-016-0212-y)
Supplement: Supplementary file 1 — 10.1186/s12951-016-0212-y Characterization of functionalized nanoparticles. Figure S2. Zeta potential versus pH of AGuIX (black) and AGuIX@PEG (red). Figure S3. Reaction scheme for the synthesis of AGuIX@PEG@Peptide@Cy5.5. Figure S4. Chromatograms obtained with at UV-Visible absorption at λ =295 nm of the AGuIX@PEG, AGuIX@PEG@LPFFD and AGuIX@PEG@KLVFF . Elementary Analyses of grafted nanoparticles: Table S1. Molar ratio deduced from experimental weight percentage; Table S2. Elementary analyses given in weight percent of element in the compound; Table S3. Comparison of the primary amine content evaluated by elementary analysis and TNBS assays. These results allow assessing the molecular formula obtained by elementary analysis. Figure S5. Standard curves of amine quantification in APTES/TEOS equimolar mixture by TNBS assays. Figure S6. The cyanine 5.5 presents characteristic absorption and emission wavelength λexc max Cy5.5 = 675 nm and λem max Cy5.5 = 692 nm. Table S4. The yield of the grafting was calculated thanks to the comparison of the cyanine fluorescence and the DOTA(Gd3+) chelate before and after removal of the ungrafted dye by tangential purification. Figure S7. Effect of nanoparticles on cell viability. Figure S8. PIB derivative synthesis details. Figure S9. Control of the non-binding of Cy5.5 dye to amyloid plaque in brain section of AD mouse model. [file 12951_2016_212_MOESM1_ESM.docx]

**Additional file:**

Figure S1: Characterization of functionalized nanoparticles.

Figure S2: Zeta potential versus pH of AGuIX (black) and AGuIX@PEG (red).

Figure S3: Reaction scheme for the synthesis of [AGuIX@PEG@Peptide@Cy5.5](mailto:AGuIX@PEG@Peptide@Cy5.5).

Figure S4: Chromatograms obtained with at UV absorption at λ =295 nm of the AGuIX@PEG, AGuIX@PEG@LPFFD and AGuIX@PEG@KLVFF.

Elementary Analyses of grafted nanoparticles: TableS1 . Molar ratio deduced from experimental weight percentage; Table S2: Elementary analyses given in weight percent of element in the compound; Table S3 : Comparison of the primary amine content evaluated by elementary analysis and TNBS assays. These results allow assessing the molecular formula obtained by elementary analysis.

Figure S5: Standard curves of amine quantification in APTES/TEOS equimolar mixture by TNBS assays.

Figure S6: The cyanine 5.5 presents characteristic absorption and emission wavelength λex.max cy5.5 = 675 nm and λem.max cy5.5 = 692 nm.

Table S4: The yield of the grafting was calculated thanks to the comparison of the cyanine fluorescence and the DOTA(Gd3+) chelate before and after removal of the ungrafted dye by tangential purification.

Figure S7: Effect of nanoparticles on cell viability.

Figure S8: PIB derivative synthesis details.

Figure S9: Control of the non-binding of Cy5.5 dye to amyloid plaque in brain section of AD mouse model.


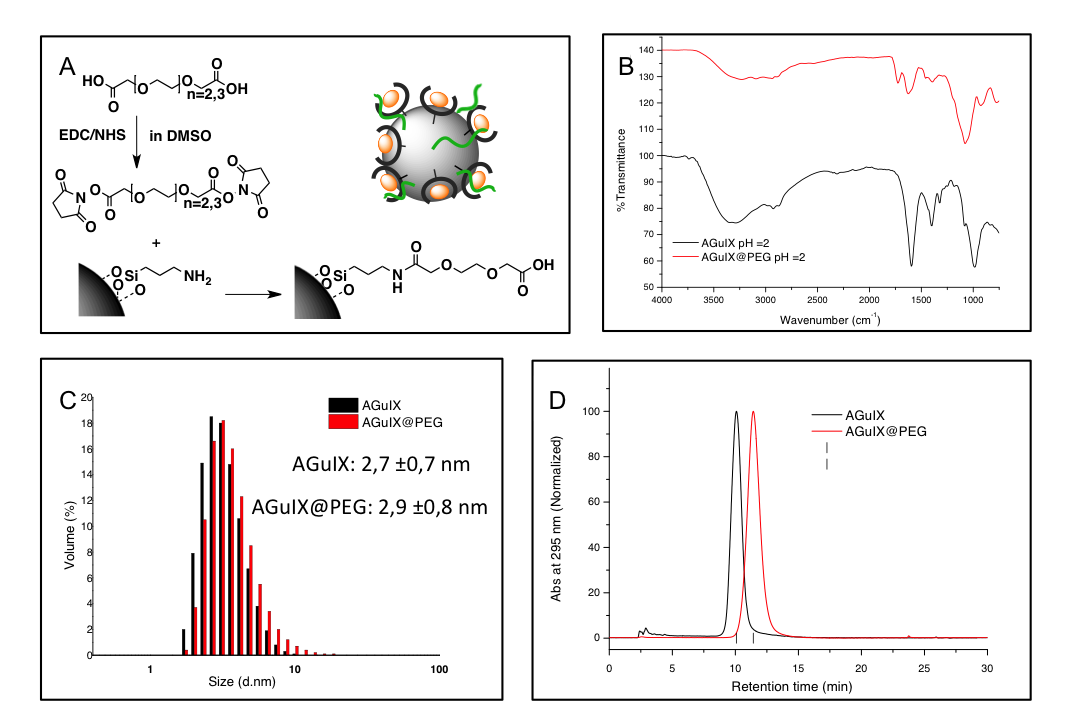


**Figure S1: Characterization of functionalized nanoparticles**:

(A) Reaction scheme of AGuIX Nps pegylation. (B) FTIR spectrum of AGuIX (black) and AGuIX@PEG (red) dried at pH=2. The FTIR spectrum of the dry products were acquired on IR-Affinity-1, Shimadzu^®^ with an ATR platform by applying attenuated total reflection Fourier transform infrared (ATR-FTIR) spectroscopy from 800 to 4000 cm^-1^. The Nps suspensions were dried at 80 °C overnight after adjusting the pH at 2 with HCl 0.12 M. The apparition of a peak at 1716 cm^-1^ (ν_c=o_ stretch) observed by FTIR is characteristic of the introduction of carboxylic acid moiety. (C) AGuIX (black) and AGuIX@PEG (red) hydrodynamic diameter measurements by DLS (λ= 532 nm); the hydrodynamic diameter (D_H_) of the AGuIX@PEG (2.9 ± 0.9 nm) showed a slight increase compared to the D_H_ of the AGuIX (2.7 ± 0.7 nm), (D) HPLC chromatograms obtained with detection by UV-Visible absorption (λ = 295 nm) for AGuIX (black) and the AGuIX@PEG (red) .The retention time shift from 10.1 min. to 11.4 min. is observed in accordance with the Nps surface modification by PEG chains.

**
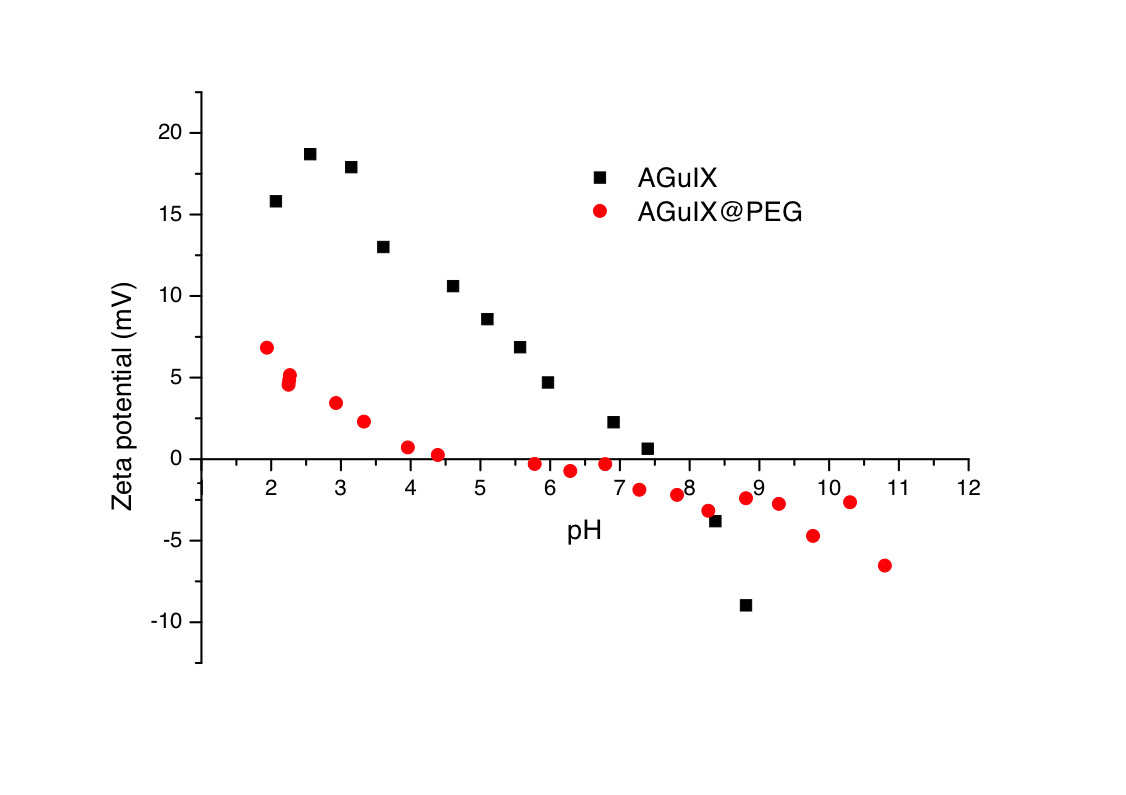
Figure S2: Zeta potential versus pH of AGuIX (black) and AGuIX@PEG (red).**

ζ-Potential measurements were performed with a Zetasizer NanoZS (laser He-Ne 633 nm) equipped with a MPT-2 autotitrator device from Malvern Instrument^®^. The colloidal solutions were firstly diluted at 10 mM of Gd^3+^ in an aqueous solution containing 5 mM of NaCl. One measurement was performed starting at pH 5.5 and going to pH 2 by 0.12M HCl addition and another one starting from pH 5.5 and going to pH 12 by 0.1M NaOH addition.

The isoelectric point of the nanoparticles slightly decreased (from pH 7.5 to pH 6) in accordance with a modification of the nanoparticles surface charge obtained by changing an amine into a carboxylic acid.

**
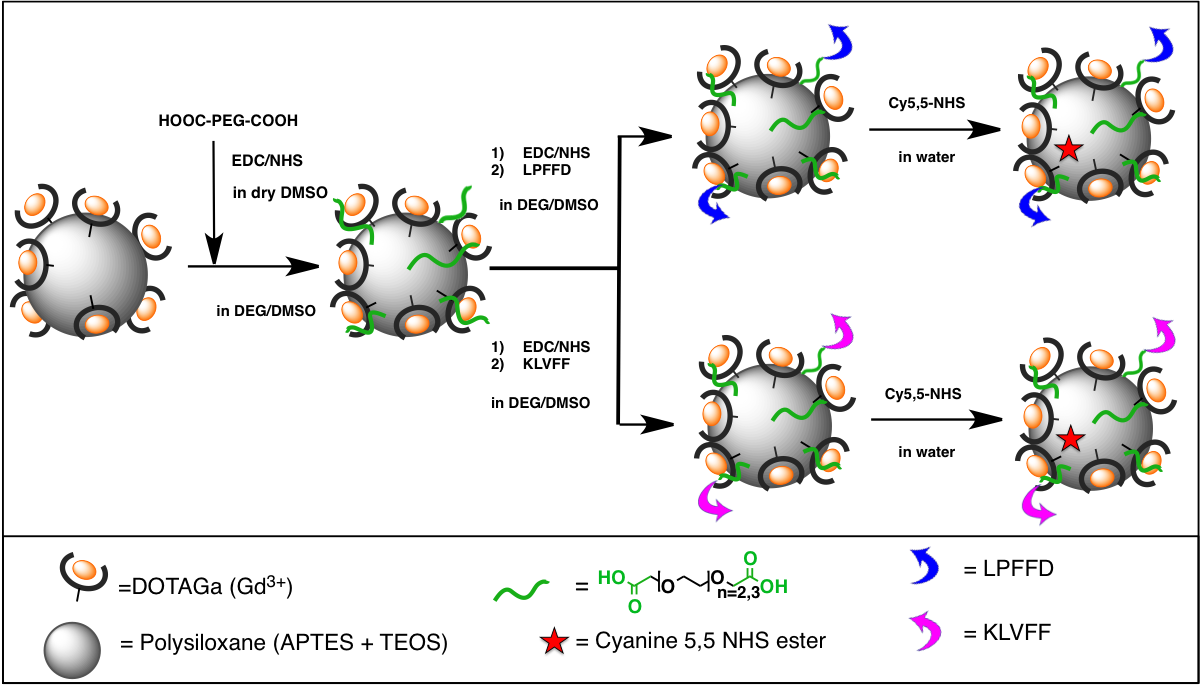
**

**Figure S3: Reaction scheme for the synthesis of AGuIX@PEG@Peptide@Cy5.5**

The AGuIX nanoparticles are very hydrophilic. On the contrary, peptides present a hydrophobic character due to their two phenylalanines residues. The coupling occurred in a DEG/DMSO mixture, allowing i) the limitation of the NHS ester hydrolysis, ii) the proper dispersion of both the nanoparticles and the hydrophobic peptides.


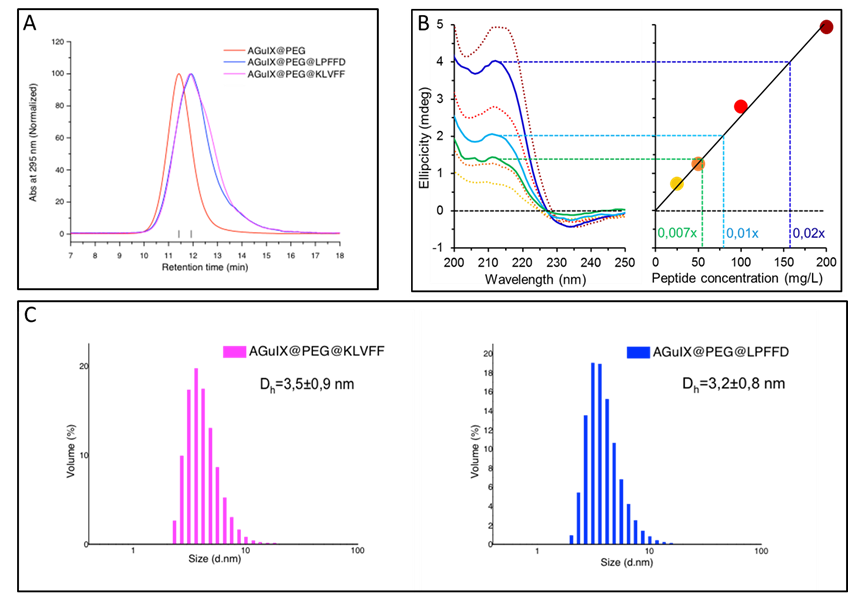


**Figure S4:** Chromatograms obtained with at UV absorption at λ =295 nm of the AGuIX@PEG(red), AGuIX@PEG@LPFFD (blue) and AGuIX@PEG@KLVFF (pink). A slight shift of the retention time confirmed the grafting (11.9 min. for both peptide vectorized nanoparticles, against 11.4 min. for the PEG-nanoparticles). Besides, the Full Width at Half Maximum (FWHM) is almost twice larger for the peptide vectorized nanoparticles (1.6 min. for the AGuIX@PEG@LPFFD and 1.8 min. for the AGuIX@PEG@KLVFF versus 1.1 min. for the AGuIX@PEG). These results suggest that the distribution of nanoparticles is wider after peptide grafting.

***Elementary Analyses :***

To evaluate the number of PEG chains grafted to the nanoparticles, elementary analyses were performed on the nanoparticles. The hypothesis that about 10 DOTA molecules are displayed on one nanoparticle was made. The molar ratio (i.e. Si/Gd, C/Gd and N/Gd) deduced from weight percentage allowed tracing back the approximate molecular formula of each nanoparticles.

|  | AGuIX | AGuIX@PEG | AGuIX@PEG@LPFFD | AGuIX@PEG@KLVFF |
| --- | --- | --- | --- | --- |
| Si/Gd | 4.9 | 5.7 | 5.6 | 5.9 |
| C/Gd | 26.6 | 33.2 | 40.8 | 40.0 |
| N/Gd | 6.5 | 7.1 | 9.0 | 8.9 |

**Table S1**: Molar ratio deduced from experimental weight percentage

Only gadolinium, silicon, carbon and nitrogen weight percentage were measured. Thus, water molecules and counter ions such as Na^+^ or OH^-^ were taken into account. The molecular formulas were deduced with an absolute error below 0.5% of the weight percentage supplied by elementary analyses (Table S2).

|  | AGuIX | | AGuIX@PEG | | AGuIX@PEG@LPFFD | | AGuIX@PEG@KLVFF | |
| --- | --- | --- | --- | --- | --- | --- | --- | --- |
|  | Exp. | Theo. | Exp. | Theo. | Exp. | Theo. | Exp. | Theo. |
| %Gd | 13.1 | 13.1 | 11.4 | 11.5 | 10.3 | 10.3 | 10.2 | 10.1 |
| %Si | 11.5 | 11.5 | 11.7 | 11.7 | 10.3 | 10.3 | 10.7 | 10.7 |
| %N | 7.6 | 7.6 | 7.2 | 7.2 | 8.3 | 8.0 | 8.1 | 7.9 |
| %C | 26.6 | 26.6 | 28.9 | 28.6 | 32.0 | 31.9 | 31.1 | 31.2 |

**Table S2**: Elementary analyses given in weight percent of element in the compound. For each sample, first the experimental weight percentages (carried out by the “Service Central d’Analyses”, CNRS and delivered with an absolute error of 0.5%), then the theoretical weight percentage deduced from the estimated molecular ratio.

| -NH_2_ per Gd | AGuIX | AGuIX@PEG | AGuIX@PEG@LPFFD | AGuIX@PEG@KLVFF |
| --- | --- | --- | --- | --- |
| Elementary analysis  TNBS quantif. | 1.45 | 1.15 | 2.2 | 2.2 |
|  | 1.55 | 1.15 | 2.2 | 2.2 |

**Table S3** : Comparison of the primary amine content evaluated by elementary analysis and TNBS assays. These results allow assessing the molecular formula obtained by elementary analysis.

Altogether, these calculations are leading to the following formulas:

AGuIX: DOTAGa(Gd)_10_(SiO_1.5_amin.opropyl)_24,5_(SiO2)_25_;

AGuIX@PEG: DOTAGa(Gd)_10_(SiO_1.5_amin.opropyl)_30_(SiO_2_)_27,3_PEG_5_.

AGuIX@PEG@LPFFD: DOTAGa(Gd)_10_(SiO_1.5_amin.opropyl)_36_(SiO_2_)_20_PEG_3,5_LPFFD_2_;

AGuIX@PEG@KLVFF: DOTAGa(Gd)_10_(SiO_1.5_amin.opropyl)_34_(SiO_2_)_25_PEG_3,5_KLVFF_2_.

Finally, these molecular formulas were assayed by measuring the number of free amines remaining in the nanoparticles thanks to TNBS assay.


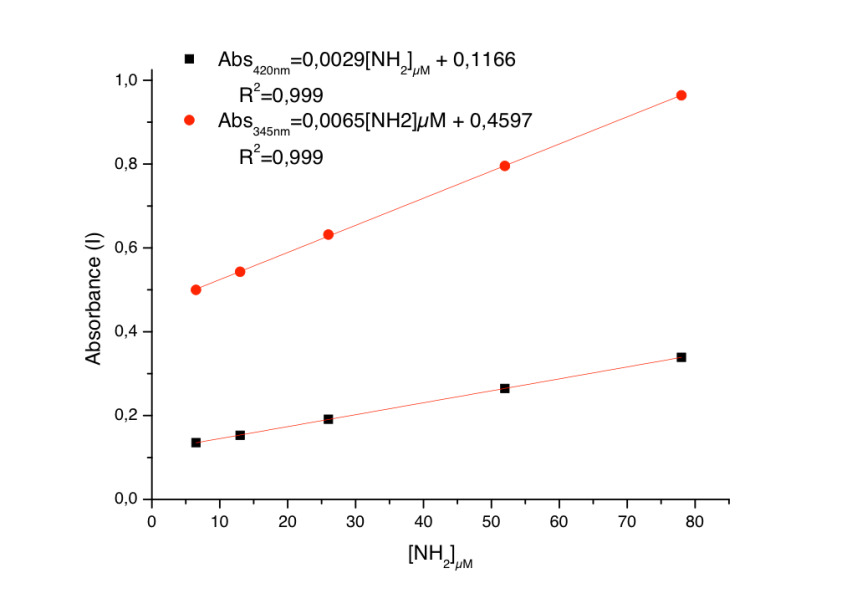


**Figure S5**: Standard curves of amine quantification in APTES/TEOS equimolar mixture by TNBS assays. i) Absorbance at 420 versus amine concentration (black), ii) Absorbance at 345 nm versus amine concentration (red). UV-Visible spectra were recorded using a UV-Vis spectrophotometer (Varian Cary50) in the range of 200 to 800 nm, with a Hellma semi-micro cell, 10 mm light path, 1400 µL, manufactured from Suprasil quartz.

The quantification of free amines in nanoparticles solutions was conducted using trinitrobenzene sulfonic acid (TNBS) assays^60^. The solutions to be assayed (10-500 µM Gd^3+^) and a negative control containing only bicarbonate buffer were performed into 1 mL of 0.1M sodium bicarbonate, pH 8.5. A volume of 500 µL of freshly prepared solution of TNBS at 2.5 mM was poured into each sample, well mixed and incubated for 2 h at 37°C, leading to the formation of a highly chromogenic derivative. The reaction was then stopped by adding 500 µL of 10%SDS and 250 µL of 1N HCl. Finally, the absorbance spectrums of the samples were measured respectively at 345 and 420 nm.

Therefore, 2,4,6-trinitrobenzene 1-sulfonic acid reacts with primary amines to form a chromogenic compound with specific absorption wavelength (345 nm and 420 nm)^61,62^. The results were compared to the number of free amine deduced from the proposed molecular formulas, as expressed in the following formulas:

AGuIX: nNH_2_ = n( SiO_1,5_amin.opropyl)– nDOTA

AGuIX@PEG: nNH_2_ = n( SiO_1,5_amin.opropyl) – nPEG – nDOTA

AGuIX@PEG@LPFFD: nb (NH_2_)= nb (SiO_1,5_amin.opropyl) – nb (DOTAGa)  – nb (PEG)

AGuIX@PEG@KLVFF: nb (NH_2_)= nb (SiO_1,5_amin.opropyl) – nb (DOTAGa)  – nb (PEG) + nb (KLVFF)

**Figure S6**: The cyanine 5.5 presents characteristic absorption and emission wavelength λ_ex.max cy5.5_ = 675 nm and λ_em.max cy5.5_ = 692 nm. The DOTAGA(Gd^3+^) chelate grafted at the Np surface present phosphorescence properties: λ_ex.max DOTAGA(Gd3+)_= 275 nm and λ_em.max DOTAGA(Gd3+)_ = 312 nm. a) Fluorescence spectra of cyanine 5.5 before and after removal of ungrafted dye (λ_exc_=675 nm and λ_em_=694 nm) respectively for AGuIX@PEG@LPFFD@Cy5.5 (a.1) and AGuIX@PEG@KLVFF@Cy5.5 (a.2). After normalization at the same DOTA(Gd) phosphorescence intensity. b) Normalized phosphorescence spectrums of DOTA(Gd) for AGuIX@PEG@LPFFD@Cy5.5 (b.1) and AGuIX@PEG@KLVFF@Cy5.5 (b.2).

|  | Cyanine 5.5  grafting yield | | Cya5.5/Gd^3+^ | Cya5.5/Np |
| --- | --- | --- | --- | --- |
| AGuIX@PEG@LPFFD@Cy5.5 | | 61 % | 0.6/1000 | 1/167 |
| AGuIX@PEG@KLVFF@Cy5.5 | | 50 % | 0.5/1000 | 1/200 |

**Table S4**: The yield of the grafting was calculated thanks to the comparison of the cyanine fluorescence and the DOTA(Gd^3+^) chelate before and after removal of the ungrafted dye by tangential purification.

**Figure S7: Effect of nanoparticles on cell viability**. The innocuousness of the grafted Nps was assessed using a neuronal cell line SH-SY5Y. The cell viability was assessed using the 3-(4,5-dimethylthiazol-2yl)-2,5-diphenyltetrazolium bromide (MTT) assay^63^. SH-SY5Y cells were cultured in DMEM/F12 medium supplemented with 10 % fetal bovine serum and 1 % penicillin -streptomycin /amphotericin B. For the assay, the cells were plated at a density of 50 000 cells/well in 200 µl of medium. Twenty four hours later, 0.5, 1, 2 or 5 mM of AGuIX@PEG@KLVFF (dark gray histograms) or AGuIX@PEG@LPFFD (gray histograms) or 1, 2 or 5mM of AGuIX@PEG (light gray histograms). Nps were added to the cells and incubated at 37 °C, 5 % CO_2_. One hour later, 10 μL of MTT solutions (12 mM) was added into each well, and the plates were incubated for another 2 h at 37°C, 5% CO_2_. The culture medium was removed, and the precipitated cells were lysed using DMSO. After 10 minutes, the purple crystals were dissolved completely; the cell viability was calculated from the absorbance signals measured by a plate reader (Tecan Infinite M200) at 540 nm. The absorbance of the wells containing medium-only, corresponding to the background, were subtracted from the value of each reading. Data are expressed in % of cell viability, each MTT analysis was carried out on triplicate samples. As expected, the amount of live cells decrease with the augmentation of nanoparticles concentration, and more than 80 % are alive when they were incubated in the presence of up to 2 mM of nanoparticles, and more than 70 % at 5 mM of nanoparticles.

**Figure S8: PiB derivative synthesis details**

Schematic synthesis of PiB derivative.

Reagents and conditions: a) Nitrobenzoyl chloride, triethylamine, THF, 0 °C, 96 %. b) Lawesson’s reagent, chlorobenzene, reflux, 85 %. c) K_3_Fe(CN)_6_, NaOH_aq_, reflux, 52 %. d) SnCl_2_, Ethanol, reflux, 92 %.

Synthesis protocol: N-(4’-methoxyphenyl)-4-nitrobenzamide (1).: To a solution of 60 mL of p-anisidine (3.08 g, 25 mmol) and dry triethylamine (3.48 mL, 25 mmol) in freshly distilled THF, was added dropwise at 0 °C, 30 mL of a solution of 4- nitrobenzoyl chloride (3.03 mL, 25 mmol) in distilled THF over 20 min.. The mixture was stirred for 1 h at 0 °C and then for 1 h at room temperature. The white precipitate was filtered and the solution was concentrated under vacuum to 30 mL. Then 50 mL of water was added and the solution was allowed to stand overnight at 4 °C. The yellow-green precipitate was filtered and dried under vacuum to give 6.53 g of compound 1 (96 %). 1H NMR (CDCl3, 25 °C, 500 MHz): δ (ppm) 3.75 (s, 3H), 6.95 (d, J =9.0 Hz, 2H), 7.67 (d, 3J = 9.0 Hz, 2H), 8.17 (d, 3J = 8.8 Hz, 2H), 8.36 (d, 3J = 8.8 Hz, 2H). 13C NMR (CDCl3, 25 °C, 126 MHz): δ (ppm) 55.2 (1C), 113.8 (2C), 122.1 (2C), 123.5 (2C), 129.1 (2C), 131.8 (1C), 140.7 (1C), 149,0 (1C), 155.9 (1C), 163.4 (1C). HRMS (ESI): m/z: calcd for C14H13N2O4: 273.08698, found 273.08697.

N-(4’-methoxyphenyl)-4-nitrothiobenzamide (2): compound 1 (6 g, 22 mmol) was dissolved in 15 mL of chlorobenzene. Then, Lawesson’s reagent (5.34 g, 13.2 mmol) was added to the solution and the mixture was refluxed 4 h. After cooling, the solution was allowed to stand overnight at 0 °C. The orange precipitate was filtered and then dissolved with acetone to separate the residue of Lawesson’s reagent. The solution was evaporated to dryness to give 5.40 g of the compound 2 (85 %). 1H NMR (DMSO, 25 °C, 500 MHz): δ (ppm) 3.37 (s, 1H), 3.78 (s, 3H), 7.02 (d, 3J = 8.8 Hz, 2H), 7.76 (d, 3J = 8.8 Hz, 2H), 7.99 (d, 3J = 8.6 Hz, 2H), 8.30 (d, 3J = 8.6 Hz, 2H). 13C NMR (DMSO, 25 °C, 126 MHz): δ (ppm) 55,3 (1C), 113.7 (C-1,5), 123.3 (2C), 125.3 (2C), 128.6 (2C), 132.6 (1C), 147,9 (1C), 148.1 (1C), 157.5 (1C), 194.1 (1C). HRMS (ESI): m/z: calcd for C14H13N2O3S : 289.06414 , found 289.06421.

2-(4’-nitrophenyl)-6-methoxybenzothiazol (3): Compound 2 (5 g, 17.3 mmol) was wetted with 3 mL of ethanol and then 15 mL of 10 % aqueous NaOH was added. The mixture was diluted with water to provide a final suspension of 5 % aqueous NaOH. The mixture was slowly added over 45 min. into a refluxed solution of potassium hexacyanoferrate(III) (22.8 g, 69.4 mmol). Then the mixture was refluxed 3 h. The brown precipitate was filtered and dried under vacuum and 100 mL of a mixture of dichloromethane-methanol (75/25 v/v) was added. The solid was filtered off and the solvent was evaporated. Then a 100 mL of a mixture of chloroform-methanol (20/80 v/v) was added to the residue and the brown-yellow precipitate was filtered to give 2.56 g of compound 3 (52 %). 1H NMR (CDCl3, 25 °C, 500 MHz): δ (ppm) 3.93 (s, 3H), 7.16 (dd, 1H, 3J = 8.9 Hz, 4J = 2.4 Hz), 7.40 (d, 1H, 4J = 2.4 Hz), 8.02 (d, 3J = 8.9 Hz, 1H, H-4), 8.22 (d, 3J = 8.9 Hz, 2H), 8.35 (d, 3J = 8.9 Hz, 2H). 13C NMR (CDCl3, 25 °C, 126 MHz): δ (ppm) 55.2 (1C), 104.2 (1C), 116.8 (1C), 124.5 (2C), 124.7 (1C), 128.0 (1C), 137.3 (1C), 139,6 (1C), 148.9 (1C), 148.9 (1C), 158.8 (1C), 162.8 (1C). HRMS (ESI): m/z: calcd for C14H11N2O3S: 287.04849, found 287.04854.

2-(4’-amin.ophenyl)-6-methoxybenzothiazol (4): To a solution of 3 (2.3 g, 8 mmol) in 120 mL of ethanol was added Sn(II) chloride dihydrate (5.87 g, 48.2 mmol) and the mixture was refluxed for 4 h. The ethanol was evaporated and the residue was dissolved in ethyl acetate. The resulting solution was washed 3 times with an aqueous solution of 1M of NaOH and the organic phase was collected and washed 2 times with 25 mL of water. The organic phase was collected, dried over MgSO4 and the solvent was evaporated. The crude product was purified by flash chromatography with dichloromethane-ethyl acetate (8/1). 1.89 g of compound 4 (92 %) was obtained. 1H NMR (CDCl3, 25 °C, 500 MHz): δ (ppm) 3.88 (s, 3H), 3.98 (s, 1H), 6.73 (d, 3J = 8.5 Hz, 2H), 7.05 (dd, 1H, 3J = 8.9 Hz, 4J = 2.4 Hz), 7.32 (d, 4J = 2.4 Hz, 1H), 7.85 (d, 3J = 8.5 Hz, 2H), 7.87 (d, 3J = 8.9 Hz, 1H). HRMS (ESI): m/z: calcd for C14H13N2OS : 257.07431, found 257.07442.


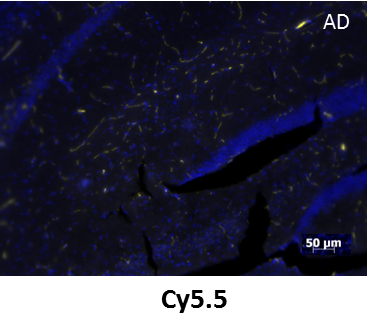


**Figure S9:** Control of the non-binding of Cy5.5 dye to amyloid plaque in brain section of AD mouse model. Pictures were photographed in the CA3-dentate gyrus area of the hippocampus.
